# Reciprocal negative feedback between Prrx1 and miR-140-3p regulates rapid chondrogenesis in the regenerating antler

**DOI:** 10.1186/s11658-024-00573-x

**Published:** 2024-04-20

**Authors:** Pengfei Hu, Guokun Zhang, Hengxing Ba, Jing Ren, Jiping Li, Zhen Wang, Chunyi Li

**Affiliations:** 1 Institute of Antler Science and Product Technology, Changchun Sci-Tech University, Changchun, China; 2 Institute of Special Animal and Plant Sciences, Chinese Academy of Agricultural Sciences, Changchun, China

**Keywords:** Chondrogenesis, Antler, miR-140-3p, Prrx1, Negative feedback

## Abstract

During growth phase, antlers exhibit a very rapid rate of chondrogenesis. The antler is formed from its growth center reserve mesenchyme (RM) cells, which have been found to be the derivatives of paired related homeobox 1 (Prrx1)-positive periosteal cells. However, the underlying mechanism that drives rapid chondrogenesis is not known. Herein, the miRNA expression profiles and chromatin states of three tissue layers (RM, precartilage, and cartilage) at different stages of differentiation within the antler growth center were analyzed by RNA-sequencing and ATAC-sequencing. We found that miR-140-3p was the miRNA that exhibited the greatest degree of upregulation in the rapidly growing antler, increasing from the RM to the cartilage layer. We also showed that Prrx1 was a key upstream regulator of miR-140-3p, which firmly confirmed by Prrx1 CUT&Tag sequencing of RM cells. Through multiple approaches (three-dimensional chondrogenic culture and xenogeneic antler model), we demonstrated that Prrx1 and miR-140-3p functioned as reciprocal negative feedback in the antler growth center, and downregulating PRRX1/upregulating miR-140-3p promoted rapid chondrogenesis of RM cells and xenogeneic antler. Thus, we conclude that the reciprocal negative feedback between Prrx1 and miR-140-3p is essential for balancing mesenchymal proliferation and chondrogenic differentiation in the regenerating antler. We further propose that the mechanism underlying chondrogenesis in the regenerating antler would provide a reference for helping understand the regulation of human cartilage regeneration and repair.

**Supplementary Information:**

The online version contains supplementary material available at 10.1186/s11658-024-00573-x.

## Introduction

Deer antlers are formed from reserve mesenchyme (RM) cells located in the antler growth center (AGC) and exhibit a very rapid rate of chondrogenesis during the growth phase [1, 2]. RM cells differentiate toward precartilage (PC) cells and further into cartilage (CA) cells, and subsequently, the cartilage tissue is replaced by bone tissue through endochondral ossification [3, 4]. Interestingly, the RM layer remains as a very thin layer throughout the period of rapid growth; this is a result of the outer sublayer of RM cells remaining mitotically quiescent, while the inner sublayer of cells is subject to intensive proliferation and then differentiation to PC cells in time, which ensures the rapid and orderly growth of deer antler [5]. Therefore, antlers can grow very rapidly because the robust RM cell proliferation and rapid differentiation into cartilage and hypertrophy of accumulated chondrocytes [6]. Thus, the antler could provide an excellent model for the study of mesenchymal differentiation toward a chondrogenic lineage [7, 8].

In previous studies, we confirmed that antler RM cells contain paired related homeobox 1 (Prrx1) positive cells [9]. Prrx1 is homeobox transcription factor that is required for mesenchymal tissue formation [10–12], and Prrx1 also has an important role in limb bud formation [13]. Studies have shown that Prrx1-positive cells are a kind of stem cells in bone [14]. Prrx1 could induce stem cell quiescence [15], and downregulation of Prrx1 reduces self-renewal of neural stem/progenitor cells [16]. Therefore, as a biomarker for stem cells, Prrx1 could maintain stem cell characteristics [17].

However, during postnatal skeletogenesis, Prrx1 has yet to be studied because Prrx1^−/−^ mouse models are lethal [10, 18]. Previous study have shown that Prrx1 expression is post-transcriptionally regulated [19], and questions remain as to how is Prrx1 regulated and how it regulates the formation and differentiation of mesenchymal tissue, especially in RM layer cells. Resolving these questions may help understanding Prrx1 functions in the rapidly growing antler and enable development of a unique model for the study of chondrogenesis.

At the post-transcriptional level, several miRNAs have been found to be key transcriptional regulators of mesenchyme differentiation toward cartilage. For example, miRNA-140 promotes bone marrow stem cells (BMSCs) differentiation toward cartilage cell [20], miR-127-5p regulates cartilage differentiation through increasing transcription factor SRY-box 9 (Sox9) expression [21], and miR-410 promotes four chondrogenic markers [collagen type II alpha 1 (Col2a1), Sox9, aggrecan (Acan), and hyaluronan synthase 2 (Has2)] expression in human cells [22]. Whereas some miRNAs inhibit chondrogenic differentiation of MSCs, for example, miR-143-3p inhibits chondrogenic differentiation through targeting bone morphogenetic protein receptor 2 (Bmpr2) in BMSCs [23], and miR-485-5p impairs BMSCs differentiation into chondrocytes through downregulating Sox9 expression [24]. Further exploration of the miRNAs related to differentiation of MSCs toward cartilaginous cells would help understand the regulation of these miRNAs and how they interact with transcription factors such as Prrx1, providing insights into the regulation of chondrogenesis.

Here, we first constructed miRNA expression profiles of the three tissue layers—RM, PC, and CA—in the AGC. We found that miR-140-3p was key miRNA during differentiation from mesenchyme to cartilage in the rapidly growing antler; then, open chromatin regions and the specific TFs binding motifs were defined by ATAC sequencing and showed that Prrx1 was a key upstream regulator of miR-140-3p, which firmly confirmed by Prrx1 CUT&Tag sequencing (CUT&Tag-seq) of RM cells. Through multiple approaches [three-dimensional (3D) chondrogenic culture and xenogeneic antler model], we confirmed that miR-140-3p and Prrx1 functioned as reciprocal negative feedback in the AGC. We propose that this negative feedback relationship plays a key role in balancing mesenchymal proliferation and chondrogenic differentiation in the growing antler and, very likely, in other cartilage model systems.

## Materials and methods

### Antler samples

Three antlers, with a growth time of 40 days calculated from shedding, were obtained from adult sika deer after anesthesia. The antler tip (AGC) was cleaned and disinfected thoroughly with alcohol swab. The AGC was sectioned along the longitudinal axis and analyzed using Paraffin and frozen sections, then carried out with HE + Alcian Blue, immunohistochemical, and immunofluorescence staining, respectively. Three tissue layers—RM, PC, and CA—were collected according to the previous reported method [5] and used for sequencing and cell isolation.

### miRNA sequencing and expression profiles analysis

Total RNA of RM, PC, and CA tissue layers were extracted using the TRIzol method, and sequencing libraries of each RNA were constructed and then sequenced on a Hiseq 2500 instrument at Biomarker Technologies Co., LTD (Beijing, China). The data analysis work was performed at Novomagic platform at Novogene Co., LTD (Tianjin, China). Differentially expressed microRNA profiles were obtained under the threshold: *p* < 0.05 and |log2(foldchange)|> 1.

### ATAC sequencing, peak calling, and enrichment of peak region-associated genes

ATAC sequencing of RM, PC, and CA tissue layers was performed using the previously reported method [25] at Novogene Co., LTD (Tianjin, China). Deer genome mCerEla1.1 (NCBI RefSeq assembly GCA_910594005.1) was used as reference for sequencing data mapping. Peak calling was performed using MACS2 v2.1.1 software, and the empirical false detection rate (FDR) < 0.05 was selected as the identified peak. The associated genes in the peak region were BLAST and annotated through searching the functional databases. The enrichment analysis of peak region-associated genes was performed using the Metascape database.

### Motif analysis of peak region enriched in the miR-140-3p promoters

Potential upstream transcription factors (TFs) of miR-140-3p were examined by analyzing the ATAC peak regions surrounding the pri-miR-140. The JASPAR CORE database (Vertebrata’s latest version) was used to identify and annotate motif of peak region enriched in the pri-miR-140 promoter, and the relative profile score threshold was set to 0.8.

### Prrx1 CUT&Tag-seq, peak calling, and annotation

First, RM cells were incubated sequentially with Prrx1 primary antibodies (Absin, China, abs134576), secondary antibody (antibodies-online, USA, ABIN101961), and pAG-Tn5 transposon. Then, Mg^2+^ was added to activate the cutting activity of Tn5 enzyme and cut off the DNA region bound to Prrx1, followed by DNA extraction and polymerase chain reaction (PCR) amplification to construct a sequencing library. Finally, library was sequenced on Illumina Novaseq platform at Novogene Science and Technology Co., Ltd (Beijing, China). The reads were mapped to the deer genome mCerEla1.1 (NCBI RefSeq assembly: GCA_910594005.1), all peak calling was performed with MACS2 (version 2.1.0), and a ChIPseeker was used to retrieve the nearest genes around the peak and annotate genomic region of the peak.

### Prediction of miR-140-3p targets

Targets of miR-140-3p were predicted by miRWalk database through searching possible binding sites of miR-140-3p within a whole gene sequence. The expression patterns of predicted target genes were further examined using RNA-sequencing (RNA-seq) data of RM, PC, and CA tissue layers in the AGC. The Gene Ontology (GO) enrichment analysis was carried out using Metascape software.

### miRNA and gene expression analysis

Total RNA was extracted from each sample, and each group has three biological replicates. miR-140-3p was reverse transcribed using stem loop primers, and the gene was reverse transcribed using random primers. The quantitative PCR reaction mixture was prepared containing reverse-transcript product, primers, and PCR Mix (TransGen, China). The primers are listed in Additional file 1: Supplementary Table 1. The PCR reaction was carried out in qTOWER3G (Analytik Jena, Germany). After reaction, data were exported analyzed using 2^−ΔΔCt^ method.

### Luciferase activity assay

The fragments of the ATAC peak regions and the mutated and deleted peak regions of miR-140-3p were inserted in the front of luciferase sequence of pGL3.0 plasmid, respectively. The constructed plasmid was co-transfected with pCDNA3.1-PRRX1 and phRL-TK plasmids into 293T cells using Lipofectamine 3000 (Invitrogen, USA). Fragments containing miRNA binding sequence and corresponding mutated sequence were amplified from Prrx1 gene, and then inserted in the behind of luciferase sequence of pmir-GLO plasmid, respectively. The constructed plasmid, miR-140-3p mimic or negative control mimic were cotransfected to 293T cells. Luciferase activity was assayed using the Spark multimode microplate reader (TECAN, Austria) after 24 h incubation and the experiment was repeated three times for each group. Primers and the detailed sequences are listed in Additional file 1: Supplementary Table 1 and Additional file 7: Supplementary Table 7, respectively.

### RM cell isolation and in vitro culture

Fresh RM tissue was cut into small fragments following the procedure reported by us [26] and then digested with collagenase mix (200 U/ml collagenase I and 150U/ml collagenase II) at 37 °C water bath for 20 min and washed twice using basic medium to remove collagenase. The digested mixture was transferred to a culture flask and fragments were made to adhere to the inner surface of the flask, cultured in a CO_2_ incubator at 37 °C.

### Immunofluorescence

RM cells immunofluorescence assay was performed in 24-well plates; the AGC tissue was first frozen and sliced, then used for immunofluorescence assay. The cells and tissues were fixed, permeabilized, blocked, and incubated overnight with primary antibodies: CD90 (1:300, proteintech, USA, 66766-1-lg), CD73 (1:1000, proteintech, USA, 12231-1-AP), Nestin (1:300, BIOSS, China, bs-0008R), CD34 (1:300, proteintech, USA, 14486-1-AP), and Prrx1 (1:200, Absin, China, abs134576). The cells were then incubated with secondary antibodies (1:50, proteintech, USA, SA00009-1, SA00009-2, SA00013-2) for 2 h, respectively. Finally, the cells and tissues were stained with 4′,6-diamidino-2-phenylindole (DAPI) for 5 min. Images were obtained and analyzed using EVOS M5000 microscope (Thermo Fisher, USA).

### Chondrogenic, osteogenic, and adipogenic differentiation

For chondrogenic differentiation, RM cells were seeded into Nunclon Sphera 3D Culture System at a density of 2 × 10^4^ cells per well with inducing reagent (OriCell, China, HUXMX-90041) without TGFβ1. The formation and growth of spheroids were analyzed at 7, 14, and 21 days using frozen section and Alcian blue staining. For osteogenic differentiation, RM cells were seeded into a 6-well cell culture plate at a density of 1 × 10^5^ cells per well with inducing reagent (OriCell, China, HUXMX-90021). The formation and growth of bone nodule were analyzed at 21 days using Alizarin Red staining. For adipogenic differentiation, RM cells were seeded into 6-well cell culture plate at a density of 1 × 10^5^ cells/well with inducing reagent (OriCell, China, HUXMX-90031). The formation and growth of lipid droplet were analyzed at 10 days using Oil Red O staining. The results were observed using Invitrogen EVOS microscope.

### Generation of miRNA sponge, overexpression, and RNAi RM cells using Lentivirus

The pCDH-CMV-miR-140-3p-sponge-EF1-CopGFP-T2A-Puro lentiviral plasmid was constructed to interfere with miR-140-3p expression. The pLVshRNA-miR-140-EGFP(2A)Puro and pCDH-CMV-Prrx1-EF1-CopGFP-T2A-Puro were constructed to increase miR-140-3p and Prrx1 expression, respectively. The pLVshRNA-Prrx1-EGFP(2A)Puro lentiviral plasmid was constructed to interfere with Prrx1 expression. Lentivirals were prepared using the constructed lentiviral plasmid. RM cells were infected with lentiviral and screened using puromycin (Solarbio, China) after 72 h of infection. The sponge sequence of miR-140-3p was designed and listed in Additional file 2: Supplementary Table 2. The primers used for plasmid construction are in Additional file 1: Supplementary Table 1. The screened cells were carried out with fluorescence detecting, qPCR, and western blot analysis, respectively.

### Xenogeneic antler mouse model treated with miRNA agomir and antigomir

Nude mice were selected for xenogeneic antler model construction, and the detailed procedure of RM tissue transplantation to nude mice is reported in our previous papers [27, 28]. Synthetic miR-140-3p agomir and antiagomir (0.15 mg per tube) were diluted with physiological saline (60 µl per tube) (20 µl per mouse) and injected into the xenogeneic antlers, with two to three injections per week for 3 weeks. The growth status of RM tissue was observed daily, and xenogeneic antler samples were taken 21 days after transplantation through anesthetizing. The xenogeneic antlers were carried out with hematoxylin and eosin (H and E) + Alcian blue, immunofluorescence staining, quantitative PCR (qPCR), and western blot analysis, respectively.

### Statistical analysis

Statistical analysis was carried out using IBM SPSS Statistics 22 software. Results were showed using prism software, and the error bars are the standard errors of the mean from three independent experiments. *p < 0.05; **p < 0.01.

## Results

### miRNA profile showed that miR-140-3p was the key positive regulator for rapid differentiation of antler mesenchyme into cartilage

**Fig. 1:**
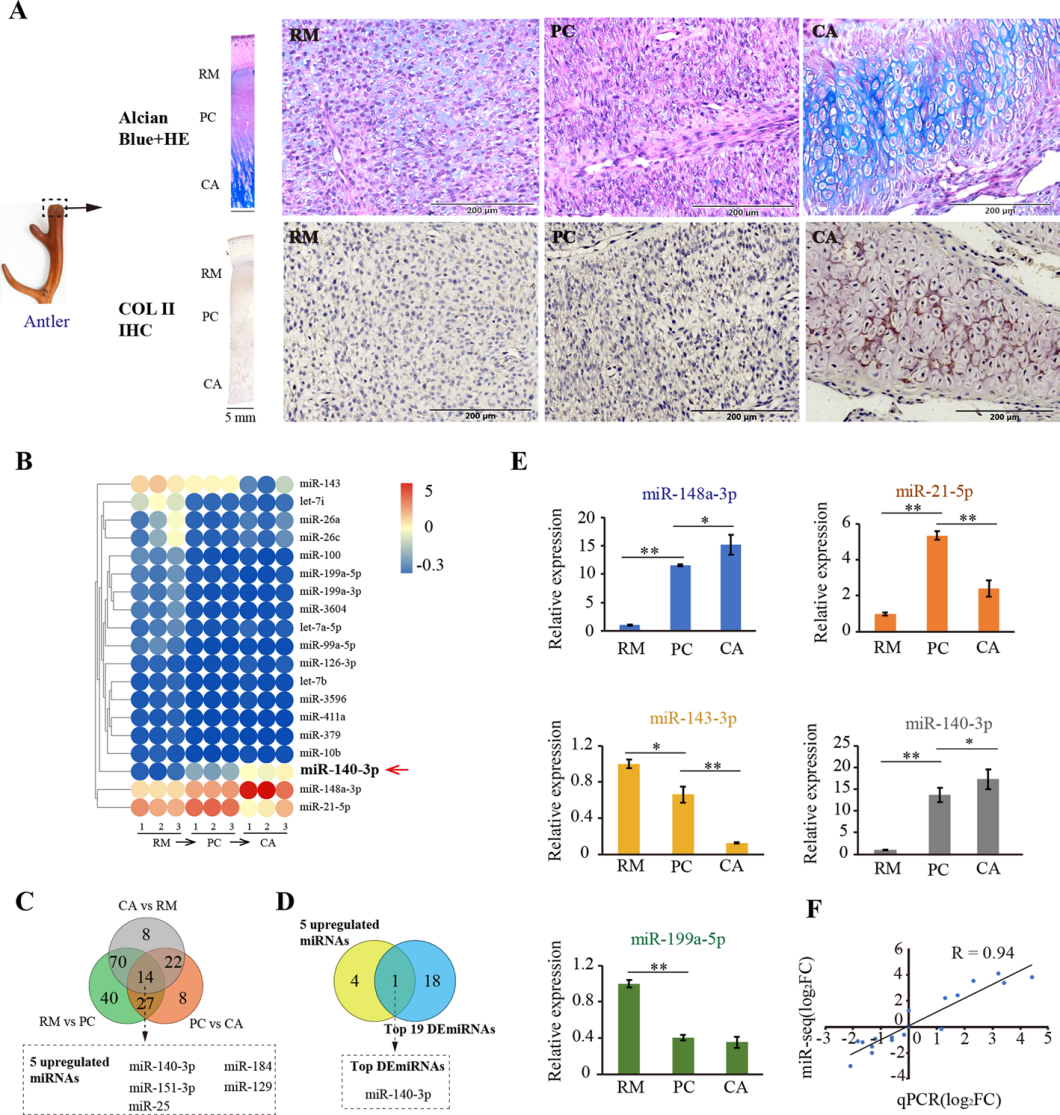
Histological characteristics and miRNA expression profiles in the antler growth center. **A** Histology of antler growth center grown for 60 days in sika deer, and the tissue layers of reserve mesenchyme (RM), precartilage (PC), and cartilage (CA) are clearly marked on tissue sections, hematoxylin and eosin (H and E) + Alcian blue, and Col II immunohistochemical staining, respectively (scale bar, 5 mm). Higher magnification version of the tissue layers of RM, PC, and CA are shown in right (scale bar, 200 μm). **B** Expression heatmap of 19 significantly differentially expressed (SDE) miRNAs between RM, PC, and CA tissue layers (TPM ≥ 5000). **C** Shared differentially expressed genes between RM, PC, and CA tissue layers. Among these miRNAs, five miRNAs were upregulated. **D** miR-140-3p was the only one of the 5 upregulated miRNAs and 19 SDE miRNAs shared between RM and PC, PC and CA, and RM and CA. **E** Expression status of five miRNAs verified using stem-loop qPCR in the RM, PC, and CA tissue layers (*n* = 3, biological replicates per group). Data are presented as the mean ± standard error. Two-tailed Student’s *t*-test was used to compare the differences between two groups. *p < 0.05; **p < 0.01. **F** Correlation analysis of stem-loop qPCR and miRNA sequencing data. The *x* axis and *y* axis denote the values of qPCR and RNA-seq, respectively

To identify the key transcription regulators during rapid chondrogenesis in the antler growth center, we sequenced and analyzed the miRNA expression profile for the three successive tissue layers, namely RM, PC, and CA (Fig. 1A). Each layer was composed of cells with relatively consistent morphology but with obvious differences between layers, with these three tissue layers representing successive stages of chondrogenic differentiation (from mesenchyme to cartilage), illustrated by Alcian blue staining and type II collagen (Col II) immunohistochemical localization (Fig. 1A).

miRNA sequencing libraries were constructed for the three tissue layers. A total of 435 known miRNAs were annotated, and their expression profiles constructed based on the transcripts per million (TPM) value of each sample; 151 significant differentially expressed (SDE) miRNAs were identified between RM and PC layers and 71 miRNAs between PC and CA layers (Additional file 3: Supplementary Table 3). In total, 19 SDE miRNAs were found and visualized using heatmap (TPM ≥ 5000, Fig. 1B). Among these SDE miRNAs, 14 were shared between RM and PC, PC and CA, and RM and CA, including 5 upregulated miRNAs (Fig. 1C). Of the 5 upregulated miRNAs and 19 SDE miRNAs, only miR-140-3p was shared (Fig. 1D), indicating that miR-140-3p was the key positive regulator of rapid differentiation from mesenchyme to cartilage in the rapid growing antler. These results were further verified using stem-loop qPCR, and the results of qPCR were consistent with the sequencing data (Fig. 1E and F).

### ATAC and CUT&Tag-seq revealed that Prrx1 was an upstream regulator of miR-140-3p in the rapidly growing antler

To explore factors that can regulate expression of miR-140-3p, the open chromatin regions and the specific TFs binding motifs surrounding the corresponding pri-miRNA loci were defined via ATAC sequencing of the RM, PC, and CA layers in the AGC.

**Fig. 2:**
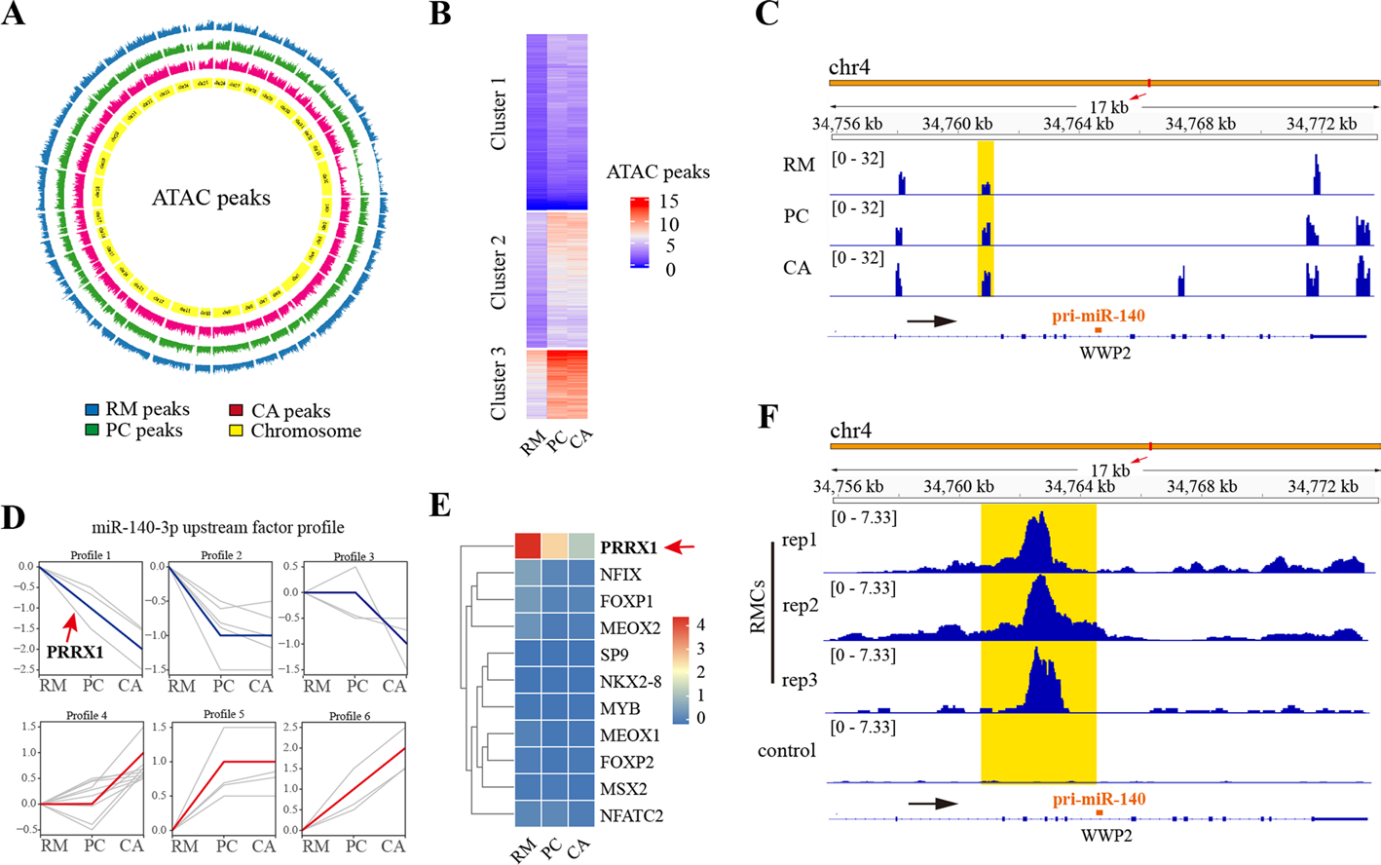
Chromatin accessibility analysis of the RM, PC, and CA tissue layers in antler growth center. **A** Genome-wide distribution of ATAC signals in the three layers. **B** Complex heatmap of ATAC signal matrix. All ATAC signals in the three layers were allocated into three clusters. **C** Genome browser view showing ATAC-seq signal around the pri-miR-140 loci. RM, PC, and CA represent three tissue layers, respectively. The yellow shadow denotes the ATAC signal that was correlated with miR-140 expression. The black arrow represents the direction of transcription. **D** Expression trend analysis of transcription factors in the upstream of miR-140-3p in the three layers. The red line represents the genes that were consistent with the expression trend of miR-140-3p. The blue line represents the genes that were opposite with the expression trend of miR-140-3p, and the line that represents Prrx1 is marked with the red arrow. **E** Expression heatmap of genes opposite with miR-140-3p; Prrx1 is marked with the red arrow. **F** Genome browser view showing CUT&Tag-seq signal around the pri-miR-140 loci. rep1, rep2, and rep3 represent three experimental repeats, respectively. RMCs represents the RM cells. The yellow shadow denotes the CUT&Tag signal that was correlated with miR-140-3p expression. The black arrow represents the direction of transcription

An average of 165,148 ATAC peaks across all chromosomes for the RM, PC, and CA layers were identified (Fig. 2A, Additional file 4: Supplementary Table 4). Genomic annotation showed that most of the ATAC peaks (57–58%) were located in distal intergenic region (Additional file 8: Supplementary Fig. 1). ATAC signals were classified into three clusters by complex heatmap analysis; the ATAC signals of PC and CA in cluster 3 were significantly enhanced compared with that of the RM layers (Fig. 2B). GO enrichment of the corresponding genes in cluster 3 showed that ossification genes were involved in bone maturation, growth plate cartilage development, and collagen fibril organization (Additional file 9: Supplementary Fig. 2). The results confirmed increasing levels of chondrogenic events in the PC and CA layers. We further found that the peaks near the pri-miRNA sequence of miR-140 (from −3434 bp to −3992 bp) (Fig. 2C), and the ATAC signal intensity was highly upregulated in the PC and CA layers compared with the RM layer, resulting in significant upregulation of miR-140 expression.

In total, 215 transcription factors were predicted to bind to the ATAC peak region (chromatin accessible region) of pri-miR-140 (relative score > 0.85). Among these, we identified 44 factors that were differentially expressed in the RM, PC, and CA layers (Additional file 5: Supplementary Table 5); of these, 17 genes expression were in accordance with miR-140-3p, while 11 genes showed the opposite trend (Fig. 2D and Additional file 5: Supplementary Table 5). According to the expression heatmap of these genes, Prrx1 was the most significant negative regulator (*p* < 0.01) (Fig. 2E).

The actual binding of Prrx1 over the entire genome in antler was directly verified using Prrx1 CUT&Tag-seq of RM cells isolated from AGC (Additional file 10: Supplementary Fig. 3). The results showed that most of the binding sites for Prrx1 were located in the promoter region (Additional file 10: Supplementary Fig. 3), and there was a strong signal near the pri-miRNA sequence of miR-140 (Fig. 2F), confirming that Prrx1 could bind to the regulatory regions of miR-140-3p. Although the peaks identified by ATAC-seq and Cut&Tag-seq do not completely overlap, which might be due to the differences in the chromatin open region under different physiological states, they were both located in the regulatory region of miR-140, further proving that Prrx1 had a regulatory effect on miR-140-3p at both tissue and cellular levels.

### Transcriptome and dual luciferase assay demonstrated that Prrx1 and miR-140-3p formed a reciprocal negative feedback relationship

To reveal biological functions of miR-140-3p during increased expression in the PC and CA layers, some potential miR-140-3p targets were predicted, and the expression characteristics of miR-140-3p targets were examined using RNA-seq data from the growing antler and sika deer tissues (NGDC accession number CRA002054).

**Fig. 3:**
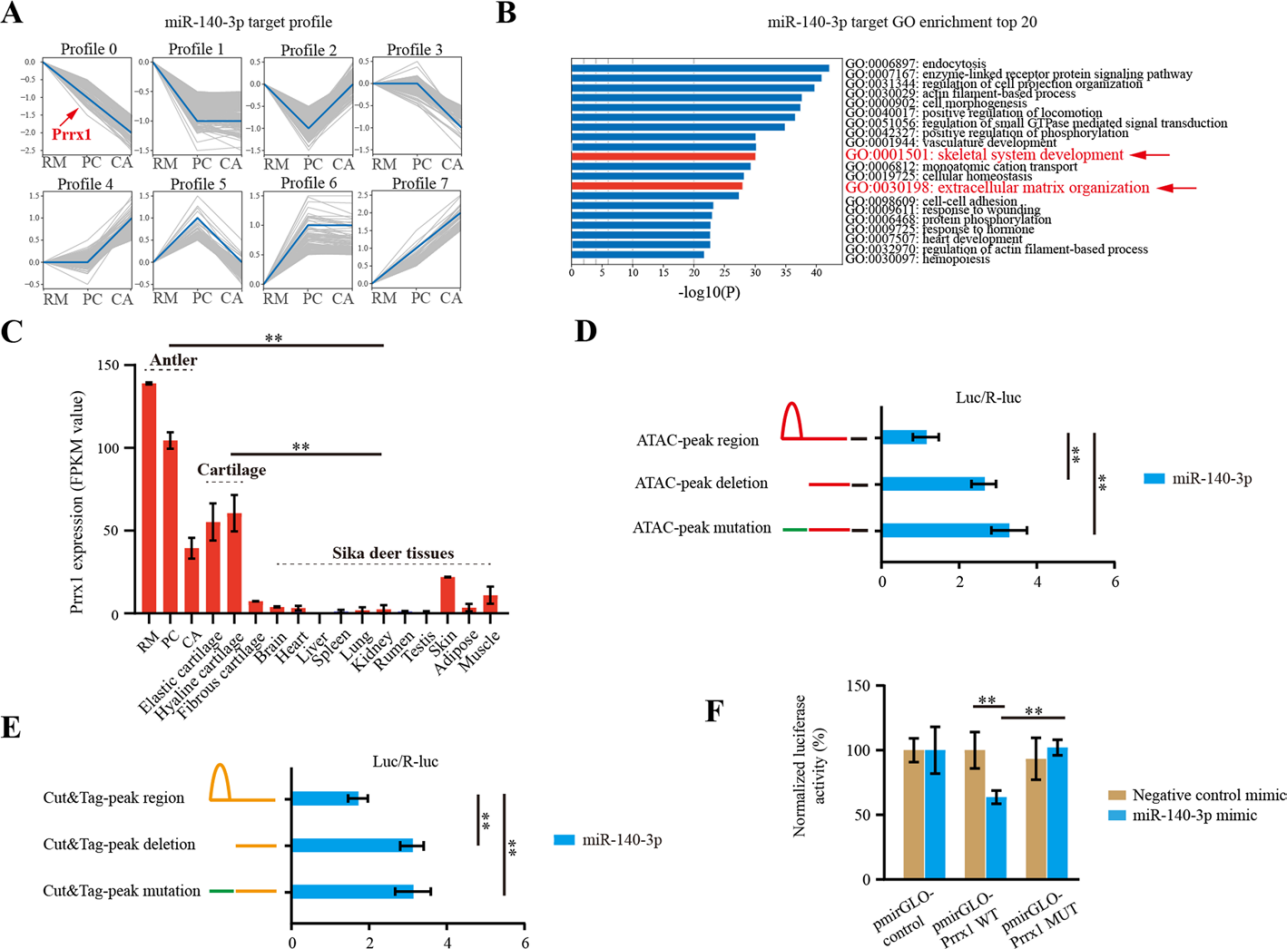
Transcriptome and dual luciferase assay of Prrx1 and miR-140-3p. **A** Expression trend analysis of target genes of miR-140-3p in the RM, PC, and CA tissue layers. **B** GO enrichment analyses of target genes of miR-140-3p. The red arrows represent the most relevant GO terms for antler growth. **C** Prrx1 expression in the different tissues of sika deer. The values were calculated using public data (accession number CRA002054, publicly accessible at https://ngdc.cncb.ac.cn/gsa). *n* = 3 biological replicates per group. Data are presented as the mean ± standard error. Two-tailed Student’s *t*-test was used to compare the differences between two groups. *p < 0.05; **p < 0.01. **D** Dual-luciferase reporter assay of the binding ability of Prrx1 to the upstream regulatory region of pri-miR-140 identified by ATAC-seq. The red line represents the ATAC peak with Prrx1 binding sites and the nearby regions. The green line represents the ATAC peak with mutant Prrx1 binding sites. The black line represents the putative promotor region. **E** Dual-luciferase reporter assay of the binding ability of Prrx1 to the upstream regulatory region of pri-miR-140 identified by CUT&Tag-seq. The yellow line represents the CUT&Tag peak with Prrx1 binding sites and the nearby regions. The green line represents the CUT&Tag peak with mutant Prrx1 binding sites. These fragments in **D** and **E** were inserted into the front of the luciferase sequence of the pGL3-basic vector in the order indicated in the figure. The relative luciferase activities were measured after cotransfection of these various constructs to 293T cells (*n* = 3 biological replicates per group). Data are presented as the mean ± standard error. Two-tailed Student’s *t*-test was used to compare the differences between two groups. *p < 0.05; **p < 0.01. **F** Dual-luciferase reporter system analysis of the interactions between miR-140-3p and Prrx1. The fragments of putative binding sites [pmirGLO-Prrx1 wild type (WT)] and the corresponding mutated binding sites [pmirGLO-Prrx1 mutated (MUT)] in the 3′ UTRs of Prrx1 gene were selected for plasmid construction. The relative luciferase activities were measured after cotransfection of 293T cells with pmirGLO-Prrx1 constructs that contained putative binding sites or the corresponding mutated binding sites in the 3′-UTRs and either miR-140-3p mimics or negative control for 24 h (*n* = 3 biological replicates per group). Data are presented as the mean ± standard error. Two-tailed Student’s *t*-test was used to compare the differences between two groups. *p < 0.05; **p < 0.01

In total, 2984 of miR-140-3p targets were obtained (Additional file 6: Supplementary Table 6), and their expression trends in RM, PC, and CA layers were visualized (Fig. 3A). GO enrichment analyses of these target genes found that the skeletal system development and extracellular matrix organization were significantly enriched (Fig. 3B), as well as ossification and negative regulation of cell differentiation (Additional file 11:Supplementary Fig. 4), further supporting the hypothesis that miR-140-3p is a key regulator in antler chondrogenesis. Interestingly, Prrx1 was one of the miR-140-3p target genes validated by RNAhybrid 2.2 (Additional file 7: Supplementary Table 7). Characteristics of Prrx1 expression in the different tissues showed that Prrx1 was highly expressed in antler and cartilage, and there was a decrease in expression of Prrx1 from RM to CA (Fig. 3C), while miR-140-3p showed an increase in expression. Based on the above observations, we hypothesized that miR-140-3p and Prrx1 formed a reciprocal negative feedback relationship in the antler growth center.

We have found support for this hypothesis through an approach using dual luciferase assay. First, when ATAC or Cut&Tag peak regions of miR-140-3p were deleted or mutated, the activity of luciferase was significantly increased compared with the wild type group after Prrx1 transfection (Fig. 3D and E), indicating that Prrx1 had the ability to bind to the open chromatin region of miR-140-3p and then inhibit their expression. These results provided support for our bioinformatics analysis that showed that Prrx1 was a transcriptional inhibitor of miR-140-3p. Moreover, we confirmed that Prrx1 was also a miR-140-3p target, and after adding miR-140-3p mimic, the fluorescence signal of Prrx1 was significantly reduced compared with the control and mutated group (Fig. 3F). So, it could be demonstrated that there was a mutual inhibition of expression between miR-140-3p and Prrx1, thus forming a reciprocal negative feedback relationship.

### Reciprocal negative feedback relationship between Prrx1 and miR-140-3p verified through increased/reduced expression analyses using the RM cells

To further determine regulatory relationship between Prrx1 and miR-140-3p in antler chondrogenic process, each tissue layer of the deer antler growth center was precisely localized, and RM cells were then isolated and cultured in vitro.

**Fig. 4:**
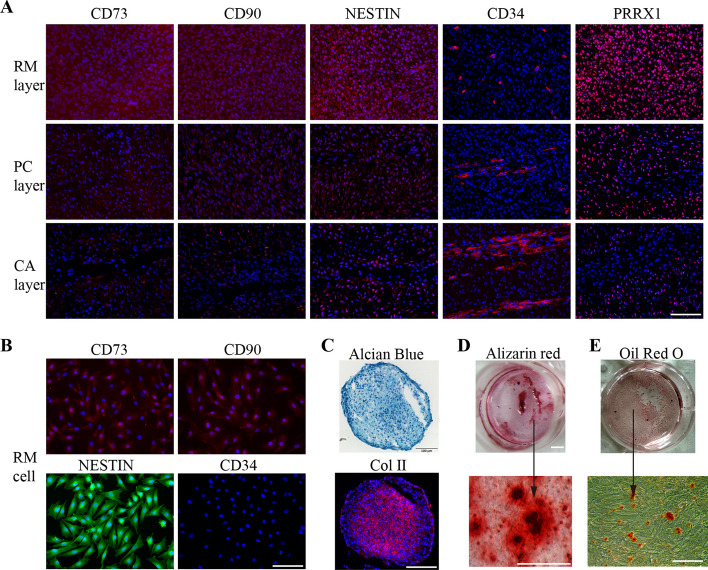
Characteristics of tissue layers of the antler growth center and isolated RM cells. **A** Immunofluorescence assay of CD73, CD90, Nestin, CD34, and Prrx1 (red color) in the RM, PC, and CA layers, respectively. **B** Third passage of cultured cells from the RM layer were identified using mesenchymal stem cell markers: CD73 and CD90 (red), Nestin and CD34 (green), respectively; nuclei were stained blue with DAPI. **C** Chondrogenic differentiation of RM cells, Alcian blue, and Col II immunofluorescence staining. **D** Osteogenic differentiation of RM cells with Alizarin red staining. **E** Adipogenic differentiation of RM cells with Oil Red O staining (scale bars, 125 μm and magnification ×200)

Frozen section-immunofluorescence staining of RM, PC, and CA layers showed that majority of cells in the RM layer expressed mesenchymal stem cell markers (CD73, CD90, and Nestin), while few hematopoietic stem/progenitor cells were found (CD34 positive) (Fig. 4A). Prrx1 was highly expressed in the RM layer, and gradually decreased as the RM cells differentiated toward PC and CA cells (Fig. 4A). The cells from the RM layer were isolated and the cell morphology was found to be relatively uniform and expressed mesenchymal stem cell markers (CD73, CD90, and Nestin) but did not express hematopoietic stem/progenitor cell markers (CD34) (Fig. 4B). The RM cells were found to exhibit pluripotency with chondrogenic, osteogenic, and adipogenic differentiation ability (Fig. 4C–E).

**Fig. 5:**
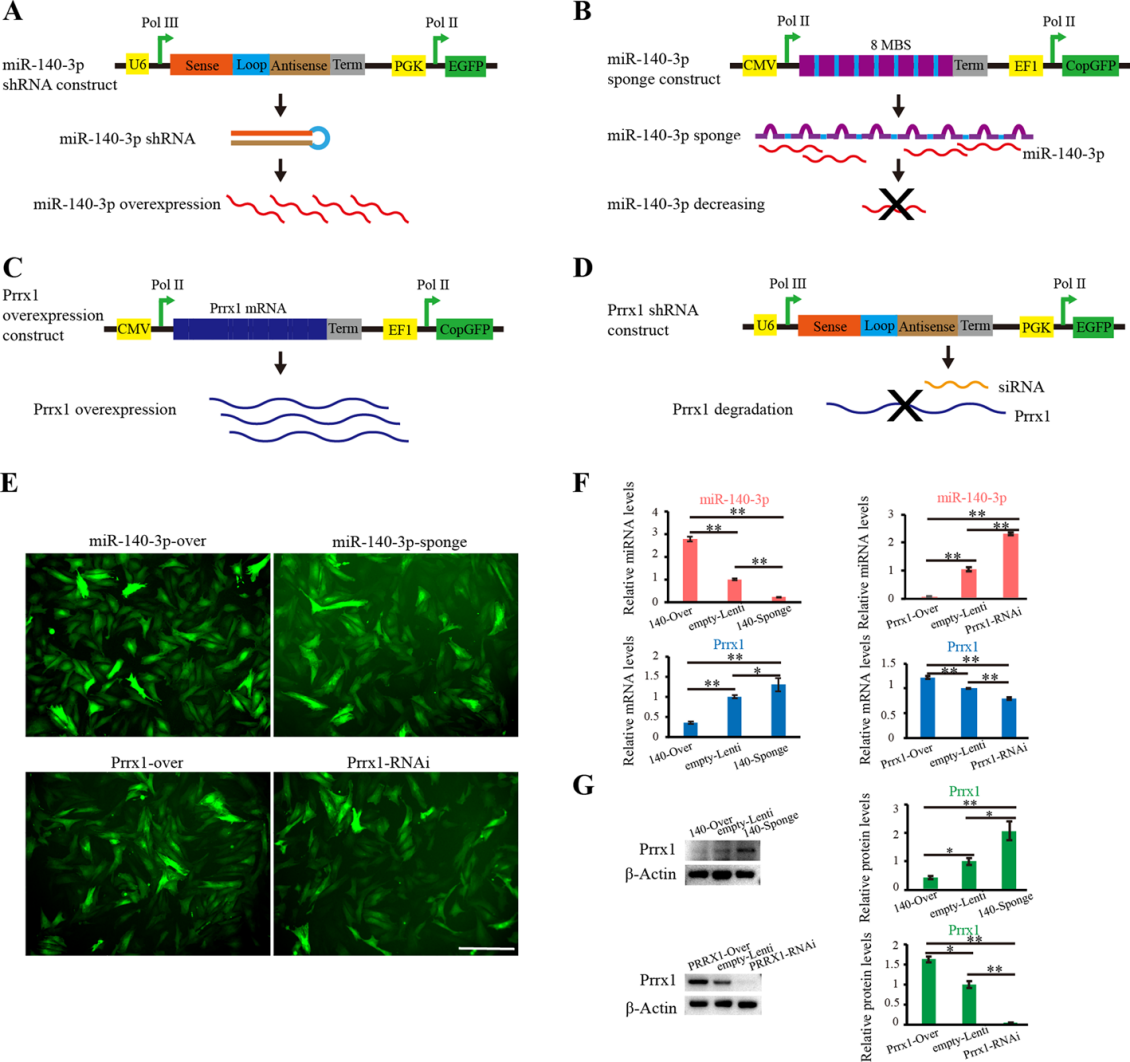
Prrx1 and miR-140-3p formed reciprocal negative feedback verified through over/reduced expression analyses in the RM cell lines. **A** Designed miR-140-3p overexpression vector. **B** Designed miRNA inhibition (sponge) vector of miR-140-3p. **C** Designed Prrx1 overexpression vector. **D** Designed Prrx1 RNAi lentivirus vector. **E** RM cell lines with overexpression or downregulation of miR-140-3p and Prrx1 (scale bars, 125 μm). **F** miR-140-3p, Prrx1 mRNA, and protein patterns via qPCR and western blot after overexpression or downregulation of miR-140-3p and Prrx1 in the RM cells (*n* = 3 biological replicates per group). Data are presented as the mean ± standard error. Two-tailed Student’s *t*-test was used to compare the differences between two groups. *p < 0.05; **p < 0.01

The miR-140 sponge and Prrx1 RNAi lentivirus vectors, as well as miR-140 and Prrx1 overexpression vectors, were designed (Fig. 5A–D). After virus infection and puromycin screening of RM cells, increased and reduced expression profiles of miR-140-3p and Prrx1 were obtained (Fig. 5E). We found that miR-140-3p overexpression resulted in downregulation of Prrx1, while interference with miR-140-3p caused an upregulation of Prrx1 (Fig. 5F). Likewise, Prrx1 overexpression resulted in miR-140-3p downregulation, while interference with Prrx1 resulted in upregulation of miR-140-3p (Fig. 5F). Prrx1 protein data were correlated with the qPCR data (Fig. 5G). The results further convinced that Prrx1 and miR-140-3p formed a negative feedback relationship at cellular level.

### Effects of reciprocal negative feedback between Prrx1 and miR-140-3p on rapid chondrogenic differentiation of RM cells

To further determine the roles of miR-140-3p and Prrx1 in chondrogenic differentiation of RM cells and their regulatory relationship, we conducted chondrogenic induction experiments on miR-140-3p and Prrx1 with increased/reduced expression RM cell lines.

**Fig. 6:**
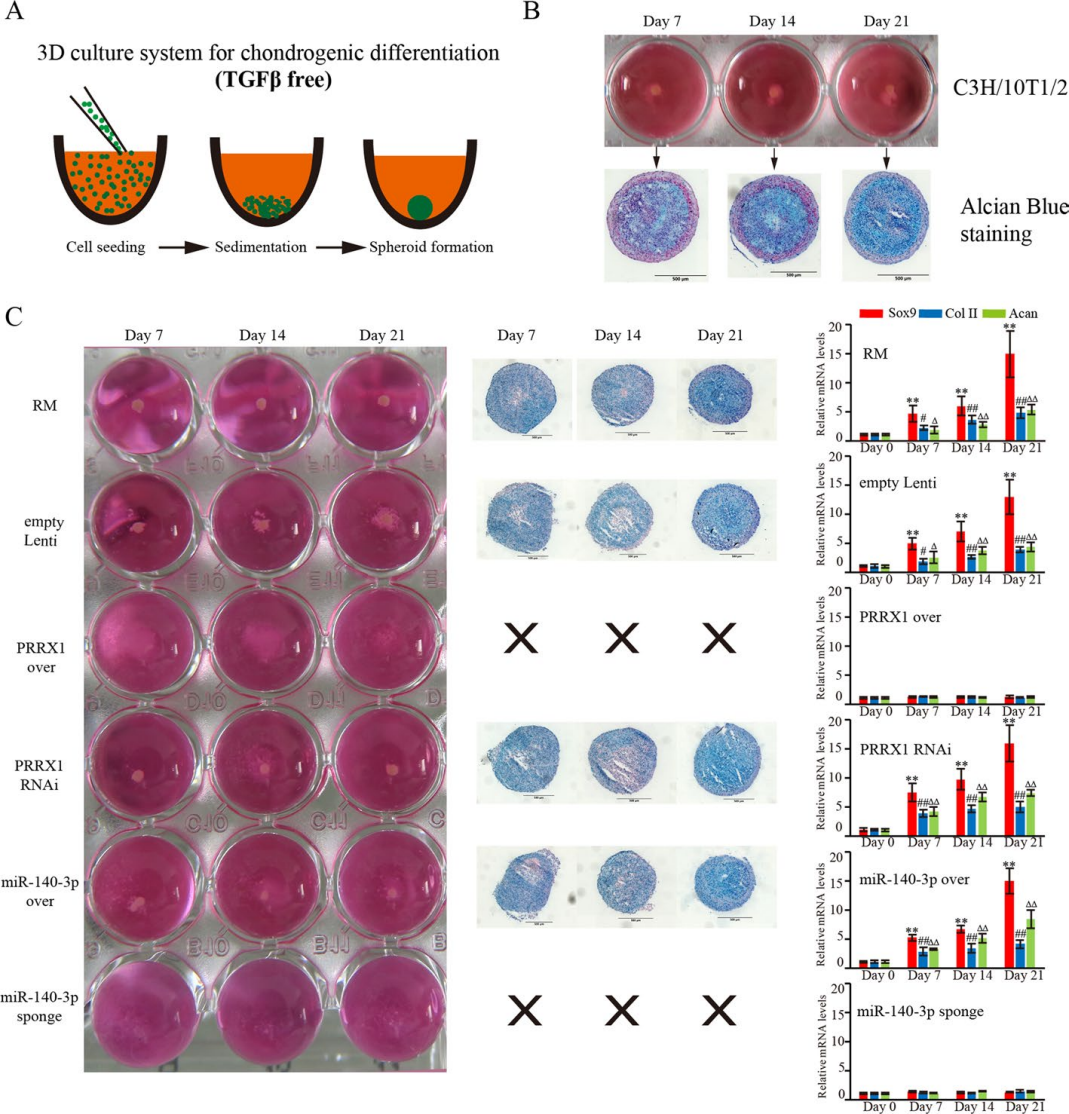
Effects of negative feedback between Prrx1 and miR-140-3p on rapid chondrogenic differentiation of the RM cells. **A** 3D culture system for the induction of chondrogenic differentiation of RM cells. **B** Chondrogenic differentiation of C3H/10T1/2 in our established 3D culture system. **C** Chondrogenic potential of the RM cells overexpressed with miR-140-3p/Prrx1 or downregulated expression of miR-140-3p/Prrx1, compared with the blank or empty lentivirus infection groups. Chondral spheroids were confirmed to have chondrocyte characteristics using Alcian blue staining, Sox9, Col II, and Acan. Expression in each group was examined using qPCR (*n* = 3 biological replicates per group). Data are presented as the mean ± standard error. Two-tailed Student’s *t*-test was used to compare the differences between two groups. The red columns represent the Sox9 gene. *p < 0.05, **p < 0.01 versus the day 0 group. The blue columns represent Col II gene, # *p* < 0.05, ## *p* < 0.01 versus the day 0 group. The green columns represent Acan gene, ∆ *p* < 0.05, ∆∆ *p* < 0.01 versus the day 0 group

First, we established a 3D culture system for inducing chondrogenic differentiation of RM cells. This system utilized the method of reducing cell attachment and adhesion to promote cell aggregation and differentiation into cartilage (Fig. 6A). We successfully performed chondrogenic differentiation of C3H/10T1/2 (Fig. 6B) and RM cells (Fig. 6C) using this system, avoiding the use of exogenous inducers such as TGFβ1.

We found that the chondrogenic ability of RM cells was enhanced with miR-140-3p upregulation or Prrx1 downregulation, but the chondrogenic abilities were not significantly different from control group, with all three groups forming similar chondroid spheroids, as evidenced by Alcian blue staining (Fig. 6C). Gene expression detection of chondroid spheroids showed that chondrogenic markers Sox9, Col II, and Acan were all upregulated (Fig. 6C). RM cells with Prrx1 upregulation or miR-140-3p downregulation formed loose cell aggregations and failed to form spheroids. Sox9, Col II, and Acan were all in low expression levels (Fig. 6C). The above results demonstrate that miR-140-3p can enhance chondrogenic marker gene expression through inhibition of Prrx1 and, in turn, promote RM cell chondrogenic differentiation.

### Effects of reciprocal negative feedback between Prrx1 and miR-140-3p on rapid chondrogenesis of xenogeneic antlers

To further determine the effects of negative feedback between Prrx1 and miR-140-3p on antler chondrogenesis, an in vivo experiment using a nude mouse xenogeneic antler model was performed.

**Fig. 7:**
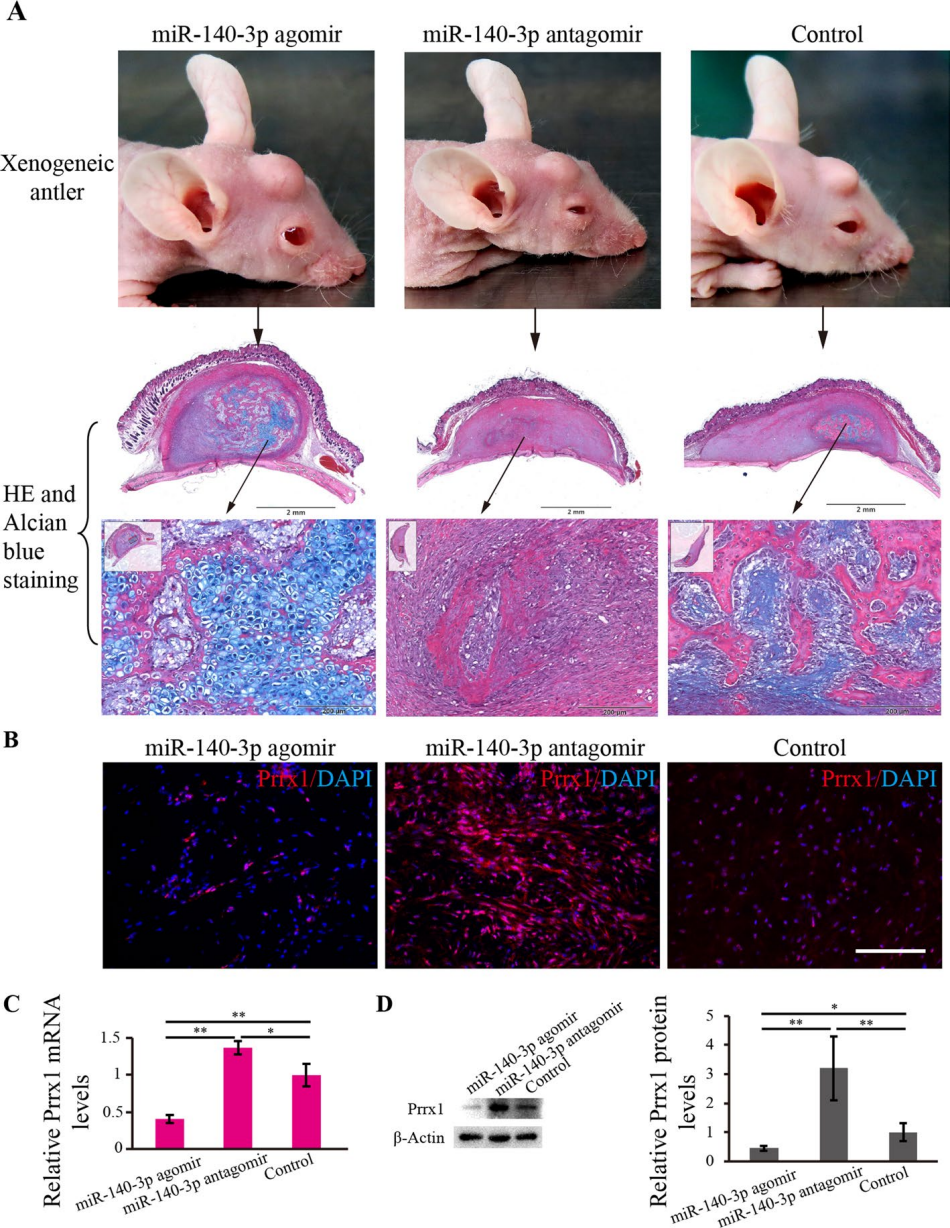
Effects of negative feedback between Prrx1 and miR-140-3p on the rapid growth and chondrogenesis of xenogeneic antlers. **A** RM layer tissue was transplanted into the area of the forehead of nude mice, and the xenogeneic antlers were formed gradually. Histological sections and H and E + Alcian blue staining of different xenogeneic antler groups showed that overexpression of miR-140-3p (miR-140-3p agomir group) resulted in the formation of large chondrocyte clusters, while inhabitation of miR-140-3p (miR-140-3p antagomir group), the majority of the formed xenogeneic antler was composed of fibrous connective tissue. **B** Frozen section and Prrx1 immunofluorescence analysis of isolated xenogeneic antlers. **C** Relative Prrx1 mRNA levels of isolated xenogeneic antlers were detected using qPCR (*n* = 3 biological replicates per group). **D** Relative Prrx1 protein levels of isolated xenogeneic antlers were detected using western blot (*n* = 3 biological replicates per group). Data are presented as the mean ± standard error. Two-tailed Student’s *t*-test was used to compare the differences between two groups. *p < 0.05; **p < 0.01

The results showed that after transplanting the tissue of RM layer subcutaneously on the forehead area of nude mice, xenogeneic antlers formed gradually (Fig. 7A), and an additional miR-140-3p injection resulted in a higher growth rate of xenogeneic antlers after 21 days of treatment. In contrast, xenogeneic antlers were significantly smaller in the miR-140-3p inhibition group than those of overexpression and control groups (Fig. 7A). Histological sections with H and E + Alcian blue counter staining of different xenogeneic antlers convinced that miR-140-3p injection promoted the formation of larger chondrocyte clusters, while most of the tissue was found to be fibrous connective tissue when miR-140-3p was inhibited (Fig. 7A). Immunofluorescence analysis showed that Prrx1 fluorescence signal was most expressed in miR-140-3p antagomir group (Fig. 7B). Gene and protein expression analysis of Prrx1 in the isolated xenogeneic antler further supported the above results (Fig. 7C, D). Therefore, it can be concluded that downregulating Prrx1/upregulating miR-140-3p promoted growth and rapid chondrogenesis of xenogeneic antlers.

## Discussion

In this study, we first focused on miRNA expression and regulation in the antler growth center of the rapidly growing antler. Through multiple different analyses, we found that miR-140-3p was involved in the rapid chondrogenesis in growing deer antler. Previously, miR-140 was proved to be a vital factor during cartilage development [29–31]. miR-140 could be activated by Sox9 and target Sp1 to maintain chondroblast proliferation [32]. When miR-140 expression was increased in MSCs, cartilage-specific genes (Col2a1, Sox9, and Acan) were significantly upregulated and the hypertrophic chondrocyte marker gene (Col10a1) was downregulated [33], and when miR-140 expression was decreased, differentiation toward chondroblasts was inhibited [34]. It was reported that miR-140 could regulate chondrogenic differentiation through the PTHrP-HDAC4 pathway [35]. These studies had shown that miR-140 was a key regulator during MSCs differentiation toward chondroblasts, and in our study, we further demonstrated the importance of miR-140 during the rapid chondrogenesis in growing deer antler. Therefore, the mechanism through which miR-140 regulates the rapid differentiation of deer antler MSCs into chondrocytes has become the focus of this study.

**Fig. 8:**
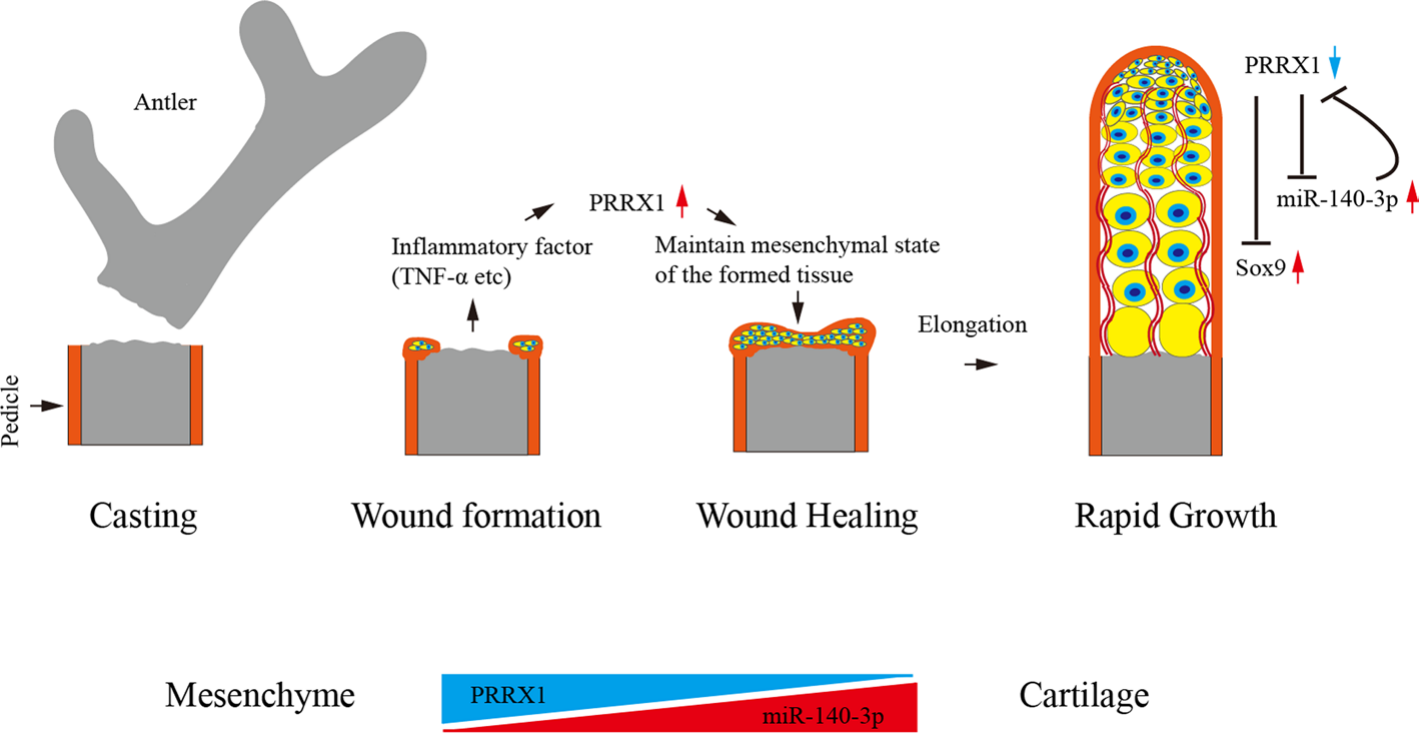
The reciprocal negative feedback between Prrx1 and miR-140-3p, as well as the effects of this feedback on rapid growth and chondrogenesis of antler

Previous studies have shown that Sox9 plays an important regulatory role in the rapid chondrogenesis of deer antler [36, 37]. The most studied interaction factor for miR-140 is Sox9; it was reported that miR-140 had close relationship with Sox9 [38]. Sox9 was considered to be a regulator of miR-140 [39], and miR-140 could promote chondrogenesis through upregulation of Sox9 [40]. Similarly, miR-140 and Sox9 might have a mutually regulatory role in the cartilage formation of deer antler inferred by bioinformatics analysis [41, 42]. However, in this study, through searching ATAC peaks in the upstream region of miR-140-3p, we did not find Sox9 binding sites. On the other hand, through transcriptome sequencing and bioinformatics analysis, we did not find that Sox9 was the target gene of miR-140-3p. So, another factor may play a mediating role between miR-140 and Sox9. Although ATAC sequencing revealed that Prrx1 can bind to the upstream regulatory region of Sox9, a relatively weak binding signal was also found through an additional Prrx1 CUT&Tag-seq. We speculate that Prrx1 may directly or indirectly inhibit the expression of Sox9, but further research is still needed to reveal the relationship between Prrx1 and Sox9. What we can confirm is that due to the increasing expression of miR-140-3p, Prrx1 was inhibited, and the maintenance of RM cell self-renewal and pluripotency was disrupted, thereby initiating the rapid process of antler RM differentiation toward chondrogenesis (Fig. 8). In this respect, revealing the specific roles and related mechanisms of miR-140-3p in rapid antler chondrogenesis could help us understand the regulatory mechanism of cartilage development in general.

Although miR-140-3p was considered as playing a key role in chondrogenesis via targeting Prrx1 in the Prrx1-positive RM cells, identification of the upstream regulatory factors for miR-140-3p is particularly important. This is crucial for explaining why deer antler has the ability to rapidly form cartilage. Due to the varying length and instability of pri-miRNA [43], the complete sequence of pri-miRNA is difficult to determine. One study has estimated that the median distance between miRNA and the promoter region was 5.5 kb [44], and it was shown that one-third of the miRNAs located in the intron had evolved to use nearby independent novel promoters independent of the host genes, with an average of 4.2 ± 3.5 kb, because the host gene promoter is much further away from miRNA, with a median distance of 57 kb [45]. This was confirmed in our study that although miR-140 is located within host gene Wwp2, it has already evolved its own promoter in nearby regions, which could facilitate faster and more effective transcription of pri-miR-140. this might be one of the reasons why deer antler has the ability to rapidly form cartilage.

In our study, ATAC sequencing was used to identify gene upstream regulatory sequences. ATAC sequencing combined with transcriptome analysis has revealed many biological phenomena, such as the identification of key driving factors related to skeletal muscle development [46], cortical neurogenesis [47], hippocampus and cerebral cortex [48], and elderly macular disease [49]. These studies indicated that ATAC sequencing is an effective method for identifying gene upstream regulatory sequences. Therefore, we adopted this method to determine the upstream regulatory regions and ultimately identified and confirmed that Prrx1 was a negative regulatory factor for both miR-140-3p. We demonstrate that ATAC sequencing is an effective method for identifying upstream regulatory factors of miRNA.

## Conclusions

The reciprocal inhibitory expression relationship between Prrx1 and miR-140-3p revealed the regulatory mechanism of deer antler differentiation from mesenchymal to cartilage. This negative feedback relationship is of great significance for maintaining mesenchymal proliferation and chondrogenic differentiation. Breaking this relationship would change the fate of antler RM cell differentiation and ultimately initiate rapid chondrogenesis. The mechanism discovered in deer antler also provides a reference point for helping understand the regulation of cartilage regeneration and repair in other model animals including humans.

### Supplementary Information

**Additional file 1: Supplementary Table 1.** Primers

**Additional file 2: Supplementary Table 2.** miRNA sponge design

**Additional file 3: Supplementary Table 3.** Identified miRNAs.

**Additional file 4: Supplementary Table 4.** Identified ATAC and CUT&Tag peaks.

**Additional file 5: Supplementary Table 5.** Identified transcription factors

**Additional file 6: Supplementary Table 6.** Target genes.

**Additional file 7: Supplementary Table 7.** Sequence information.

**Additional file 8: Supplementary Fig. 1.** Genomic annotation of the ATAC peaks. **A** RM ATAC peaks. **B** PC ATAC peaks. **C** CA ATAC peaks.

**Additional file 9: Supplementary Fig. 2.** GO enrichment of the corresponding genes in cluster 3 of ATAC peaks.

**Additional file 10: Supplementary Fig. 3.** Prrx1c CUT&Tag-seq. **A** Prrx1 CUT&Tag peak on chromosome distribution. **B** Prrx1 CUT&Tag narrow peaks plot. **C** Prrx1 CUT&Tag summits heatmap.

**Additional file 11: Supplementary Fig.4.** GO enrichment of target genes of miR-140-3p.

## Data Availability

The raw sequencing data reported in this paper have been deposited in the Genome Sequence Archive in National Genomics Data Center, China National Center for Bioinformation/Beijing Institute of Genomics, Chinese Academy of Sciences (miRNA-seq CRA014860, ATAC-seq CRA014751, and Prrx1 CUT&Tag-seq CRA014775) that are publicly accessible at https://ngdc.cncb.ac.cn/gsa.

## Abbreviations

Prrx1: Paired related homeobox 1
RM: Reserve mesenchyme
ATAC: Assay for transposase accessible chromatin
AGC: Antler growth center
PC: Precartilage
CA: Cartilage
MSCs: Mesenchymal stem cells
Sox9: Transcription factor SRY-box 9
Col2a1: Collagen type II alpha 1
Acan: Aggrecan
Has2: Hyaluronan synthase 2
Bmpr2: Bone morphogenetic protein receptor 2
FDR: False detection rate
TSS: Transcription start site
GO: Gene ontology
TPM: Transcripts per million
SDE: Significant differentially expressed
TFs: Transcription factors
