# Supplementary material for: Reciprocal negative feedback between Prrx1 and miR-140-3p regulates rapid chondrogenesis in the regenerating antler
Source: Cell Mol Biol Lett. 2024 Apr 20;29:56. doi: 10.1186/s11658-024-00573-x (PMC11031908; doi:10.1186/s11658-024-00573-x)
Supplement: Supplementary file 2 — Additional file 2: Supplementary Table 2. miRNA sponge design [file 11658_2024_573_MOESM2_ESM.docx]

bta-miR-140: TACCACAGGGTAGAACCACGGA

Generated sponge sequence at DNA level (sense strand): 5'-TCCGTGGTAACCCCTGTGGTAAATTTCCGTGGTAACCCCTGTGGTAAATTTCCGTGGTAACCCCTGTGGTA-3'

Generated sponge sequence at DNA level (anti-sense): 5'-TACCACAGGGGTTACCACGGAAATTTACCACAGGGGTTACCACGGAAATTTACCACAGGGGTTACCACGGA-3'

Generated sponge sequence at RNA level: 5'-UCCGUGGUAACCCCUGUGGUAAAUUUCCGUGGUAACCCCUGUGGUAAAUUUCCGUGGUAACCCCUGUGGUA-3'


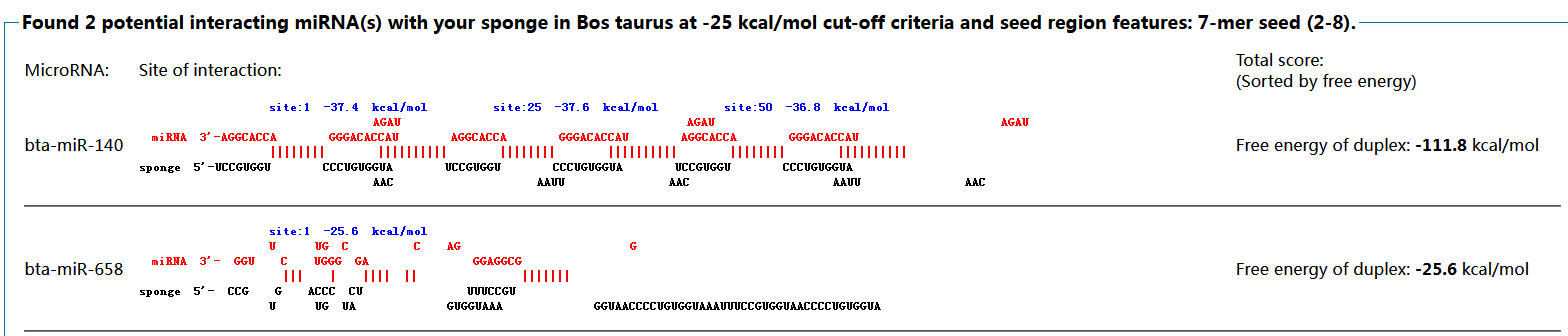


140 sponge sequence at DNA level (sense strand): 5'-GAATTCTCCGTGGTAACCCCTGTGGTAAATTTCCGTGGTAACCCCTGTGGTAAATTTCCGTGGTAACCCCTGTGGTAAATTTCCGTGGTAACCCCTGTGGTAAATTTCCGTGGTAACCCCTGTGGTAAATTTCCGTGGTAACCCCTGTGGTAAATTTCCGTGGTAACCCCTGTGGTAAATTTCCGTGGTAACCCCTGTGGTAGGATCC-3'
