# Supplementary material for: Reciprocal negative feedback between Prrx1 and miR-140-3p regulates rapid chondrogenesis in the regenerating antler
Source: Cell Mol Biol Lett. 2024 Apr 20;29:56. doi: 10.1186/s11658-024-00573-x (PMC11031908; doi:10.1186/s11658-024-00573-x)
Supplement: Supplementary file 7 — Additional file 7: Supplementary Table 7. Sequence information. [file 11658_2024_573_MOESM7_ESM.docx]

**Upstream sequence of pri-miR-140 of deer**

CAGAAACAAGTGTTGAAAGCCGAGAAACTTTTAAGTTGGGAAAGGTGAGCGCTATCTTGCCGGCCGTCCACCTGGAGCCAATGGGTGCCCCCTTCAGTCCCTTTCAGAATCACCAGCTAGAGTTGAGTTTGCCCCTCCAGCCCATGCTTTCTCCCTCAAACAATTCAGAGCCATCGTTAGTACAGCAGTAAAGGGCCAGAGAGTAAATATTGTCCAGAGGAGAAAGTATGTCTGAGCTCACCAGACACTGCCCGTCTTGAGGCACACACTTAGGAATAATACCAGGAAGCCCACAGCCCAAGGTGCGTGTCTGCATGTATGCACAACATAGACATTTTGCTGGGATACGGGGCTTTGAGAACCAAACTTTGCGTTTAAGCAGGAAGGTACAGAGGCCCGGTGGTGCTGAGCAGAGAGGGATGAGGGAAAAGGCAGCCTCCCCCCCGGCCCCCCCAGTGACTGTGCCTGTGGGACAGGGATGCCTAGGTCGGCCGCATCCAGTGAACAGAGGACCAGAGAGCAGCCGTGATGGACTGGGGTGGGCACCAGGGTCGAGGGGAGGCTGGGCTGTGCCTCCACTTTGCACACAGCTGG~~AT~~**~~GCAATTAC~~**~~GCCTCTGGTTTCAG~~CTGGCCCGCTCCCCCTCCGTGTTGCCACACCGCCCCCTTCCCTCCAGCAAACCAACCAACCGACCAACCGAAGACGGTGCTTCATCTTGAGTTGAAAACGTGTATACCCCTCCCCTGCTCCCCAGTAGGGAAAGATTCTTTTTGTCCTTTTTTTCGCCAGTGACAGACATGAGTGAGCCAGGTTGCGCTGGTTAAACTTCTCTAACAGGGTGAGGACAGGGGCAGAGATGAAGACCAGCACCCAGGAGGTGGGAGCCAGCGGGGAGAGGACATGGGCTCAGGGTGTCTCCATCTGAGCATCCACTTTTCTCTCTCCTCCCCGGCAGAGAAGAGACAGGACAATGGACGGGTGTATTACGTGAACCACAACACACGCACGACCCAGTGGGAAGACCCTCGCACCCAGGGGTGAGGACTTAGGCCGGGAGGGCTCTGGGTGGGCCCTGTGCACCTCTGTTGGCCTGGTGGCCTCACACTGTATTTGGCCTCCGTGGCTGATGTTCTGGGACCTTTAAGCACACACAAGTGCCCCCAGACCCTCACGATATAGTGAGAGCTTGGAAGTGGCATTTTATGTTGGCCTTCCTCATGGCTGCAGCGCGGCCTGACAGGCCGGTGTTGCTGGCCCCTGCTGTACAAGCTGCTGATGAAGTGATTTCAGAAGAGTTACGTGAGAACTCCCACCGACCCCAACACTCCTTCACTACCCACCCCCCAGAAGCTAGTCTGGGATGGGGCATTTGATGTCTTGGGATTGCTGGCGGCCACTGAGGCCACGGTCATCCACCCAGGGCGGGGATTCTTTGGTCTTGGGACAGTAAATGACCTCTCCACTGCAGACCTTGGGTATTGCCAAGTACATTTCCTTGAGGCCTCTGCGGGGTCTGGGTAAAAGCAGAGAGAAGGCAGCTCCAGCCTTCTGGGGACCCCAGCCCCCTTAGCATGGAAGCCCAGAAGGTGAGATATGAGGCCAGCCTGCAGCCCCTCTCTTCCCATGTTTCCAGGATGATCCAGGAGCCGGCTCTGCCCCCAGGGTGGGAGATGAAGTACACCAGCGAGGGGGTGCGCTACTTCGTGGACCACAACACCCGCACCACCACCTTTAAGGATCCTCGCCCGGGGTTTGAGTCAGGGTAAGGACTTTGCATGTAGGGCTTCCCAGGTGGCGCTAGCGGGAAAGAATCTGCCTACCAGCGTAGGAGATGAAAGAGACATGGGTTCAATCCCTGGGTCAGGAAGATCCCCTGGAGAAGGAAATGGCAACCCACTCCAGTATTCTTGCCTGTAAAATCCCATGCACAGAGGAGCCTGGCGGACTGCAGTCCATGGGGTCACAAGACTGAGCGCCTGAGCACCAGCGGACTTTGCGCAGGTGTTAGCACAGGCCTAGTATAGCTGCTTTTTTTTTTTTTTTTTTTGTATAGCTGCTTTTACCACCCTCTAGTGGCCAGTTTTGATGCCACGCCAGTGGGGATGGGTAGGGAAGGGGCTTCTCTATTGAGACTAACACTGGTGGTCTTTCCCTAAAAGCAAGAGGTCTGGCTTTATTTCTAGATTTTCTTTTTAACCTTCAAGTCTGCAGAAGATCTTAGTGGCAGTCCCTTCCTTTCTTTTATGCTTCATTTTACTCATCTC**TAAATTAA**CAGAGCAGTAGCTTTCCTTAGGGCTAATATGTTGCCAAGTTTTGAGAAATCTCGTGGATTCCAGAACATCTTTGCTTTTCCCAGGACAAAGCAAGGTTCCCCTGGCGCCTACGACCGAAGTTTTCGGTGGAAGTATCACCAATTCCGTTTCCTCTGCCATGTGAGTTCTGGTACTGGGGCTTCCCCCTGGAGATTGGGGTTACAGAGTCATTTCATCATTTCCTAGCCTAGTTGCAGGCGTTGGAATGCCTGTGTCTTAGGTGGGAGCAGGTCACTTGTCTCACTGTGACATTGTCTTCCATTCCTAGGGGACCAAGTTCTACCCCCCAAACGTGGAGGTGGGGGGGTAGGGGAAGCCTATTCAACAGACAAGTGATCAGATTGAATAGTTACCACCAATGCCCCAATTTGAGGTCTCTGTTTTCCTTTTCTTTTCAGTCAAATGCTCTCCCCAGCCATGTGAAGATCAGTGTTTCCAGGCAGACACTTTTTGAGGATTCTTTCCAGCAGGTTAGAGAATAATCATCGAGTCTAAGACCCGGGGCAGGGAGAGGGCCCTCTGCTACCACCCTGTCTTCTCTGGTGTGGCCCTTTA

**Prrx1 binding sites of ATAC peak were validated by JASPAR**


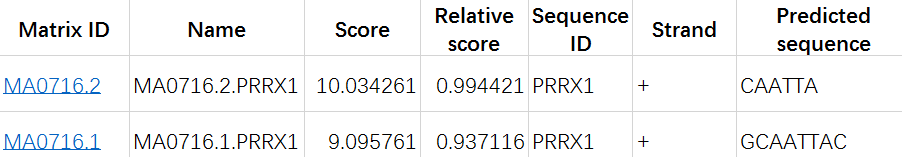


**Prrx1 binding site of Cut&Tag peak validated by JASPAR**


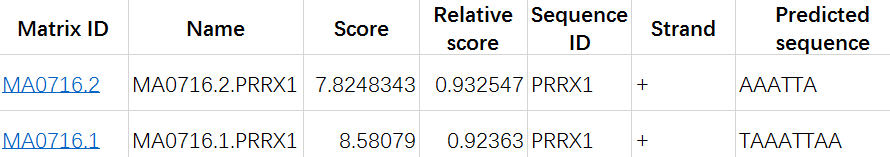


**ATAC peak with Prrx1 binding sites and the nearby regions obtained by primer 140-peak+-f and 140-peak-r**

ggtaccCACTTTGCACACAGCTGG~~AT~~**~~GCAATTAC~~**~~GCCTCTGGTTTCAG~~CTGGCCCGCTCCCCCTCCGTGTTGCCACACCGCCCCCTTCCCTCCAGCAAACCAACCAACCGACCAACCGAAGACGGTGCTTCATCTTGAGTTGAAAACGTGTATACCCCTCCCCTGCTCCCCAGTAGGGAAAGATTCTTTTTGTCCTTTTTTTCGCCAGTGACAGACATGAGTGAGCCAGGTTGCGCTGGTTAAACTTCTCTAACAGGGTGAGGACAGGGGCAGAGATGAAGACCAGCACCCAGGAGGTGGGAGCCAGCGGGGAGAGGACATGGGCTCAGGGTGTCTCCATCTGAGCATCCACTTTTCTCTCTCCTCCCCGGCAGAGAAGAGACAGGACAATGGACGGGTGTATTACGTGAACCACAACACACGCACGACCCAGTGGGAAGACCCTCGCACCCAGGGGTGAGGACTTAGGCCGGGAGGGCTCTGGGTGGGCCCTGTGCACCTCTGTTGGCCTGGTGGCCTCACACTGTATTTGGCCTCCGTGGCTGATGTTCTGGGACCTTTAAGCACACACAAGTGCCCCCAGACCCTCACGATATAGTGAGAGCTTGGAAGTGGCATTTTATGTTGGCCTTCCTCATGGCTGCAGCGCGGCCTGACAGGCCGGTGTTGCTGGCCCCTGCTGTACAAGCTGCTGATGAAGTGctcgag

**ATAC peak without Prrx1 binding sites and the nearby regions obtained by primer 140-peak--f and 140-peak-r**

ggtaccACGGTGCTTCATCTTGAGTTGAAAACGTGTATACCCCTCCCCTGCTCCCCAGTAGGGAAAGATTCTTTTTGTCCTTTTTTTCGCCAGTGACAGACATGAGTGAGCCAGGTTGCGCTGGTTAAACTTCTCTAACAGGGTGAGGACAGGGGCAGAGATGAAGACCAGCACCCAGGAGGTGGGAGCCAGCGGGGAGAGGACATGGGCTCAGGGTGTCTCCATCTGAGCATCCACTTTTCTCTCTCCTCCCCGGCAGAGAAGAGACAGGACAATGGACGGGTGTATTACGTGAACCACAACACACGCACGACCCAGTGGGAAGACCCTCGCACCCAGGGGTGAGGACTTAGGCCGGGAGGGCTCTGGGTGGGCCCTGTGCACCTCTGTTGGCCTGGTGGCCTCACACTGTATTTGGCCTCCGTGGCTGATGTTCTGGGACCTTTAAGCACACACAAGTGCCCCCAGACCCTCACGATATAGTGAGAGCTTGGAAGTGGCATTTTATGTTGGCCTTCCTCATGGCTGCAGCGCGGCCTGACAGGCCGGTGTTGCTGGCCCCTGCTGTACAAGCTGCTGATGAAGTGctcgag

**ATAC peak with mutant Prrx1 binding sites and the nearby regions obtained by primer 140-peakm-f and 140-peak-r**

ggtaccATAGCGTAGA~~GCCTCTGGTTTCAG~~CTGGCCCGCTCCCCCTCCGTGTTGCCACACCGCCCCCTTCCCTCCAGCAAACCAACCAACCGACCAACCGAAGACGGTGCTTCATCTTGAGTTGAAAACGTGTATACCCCTCCCCTGCTCCCCAGTAGGGAAAGATTCTTTTTGTCCTTTTTTTCGCCAGTGACAGACATGAGTGAGCCAGGTTGCGCTGGTTAAACTTCTCTAACAGGGTGAGGACAGGGGCAGAGATGAAGACCAGCACCCAGGAGGTGGGAGCCAGCGGGGAGAGGACATGGGCTCAGGGTGTCTCCATCTGAGCATCCACTTTTCTCTCTCCTCCCCGGCAGAGAAGAGACAGGACAATGGACGGGTGTATTACGTGAACCACAACACACGCACGACCCAGTGGGAAGACCCTCGCACCCAGGGGTGAGGACTTAGGCCGGGAGGGCTCTGGGTGGGCCCTGTGCACCTCTGTTGGCCTGGTGGCCTCACACTGTATTTGGCCTCCGTGGCTGATGTTCTGGGACCTTTAAGCACACACAAGTGCCCCCAGACCCTCACGATATAGTGAGAGCTTGGAAGTGGCATTTTATGTTGGCCTTCCTCATGGCTGCAGCGCGGCCTGACAGGCCGGTGTTGCTGGCCCCTGCTGTACAAGCTGCTGATGAAGTGctcgag

**Cut&Tag peak with Prrx1 binding sites obtained by primer Cut&Tag-peak+-f and Cut&Tag-peak-r**

ggtaccTGCTTCATTTTACTCATCTC**TAAATTAA**CAGAGCAGTAGCTTTCCTTAGGGCTAATATGTTGCCAAGTTTTGAGAAATCTCGTGGATTCCAGAACATCTTTGCTTTTCCCAGGACAAAGCAAGGTTCCCCTGGCGCCTACGACCGAAGTTTTCGGTGGAAGTATCACCAATctcgag

**Cut&Tag peak without Prrx1 binding sites obtained by primer Cut&Tag-peak--f and Cut&Tag-peak-r**

ggtaccAGGGCTAATATGTTGCCAAGTTTTGAGAAATCTCGTGGATTCCAGAACATCTTTGCTTTTCCCAGGACAAAGCAAGGTTCCCCTGGCGCCTACGACCGAAGTTTTCGGTGGAAGTATCACCAATctcgag

**Cut&Tag peak with mutant Prrx1 binding sites obtained by primer Cut&Tag-peak+-f and Cut&Tag-peak-r**

ggtaccATAGCGTAGACAGAGCAGTAGCTTTCCTTAGGGCTAATATGTTGCCAAGTTTTGAGAAATCTCGTGGATTCCAGAACATCTTTGCTTTTCCCAGGACAAAGCAAGGTTCCCCTGGCGCCTACGACCGAAGTTTTCGGTGGAAGTATCACCAATctcgag

**Deer Prrx1 mRNA sequence**

TTTCTGGTCCGAGGGAAAGGAGGAAGAAGGAGATTGTGATGGAGAAAGGGGGTCTGTAAACCGTCAGGCACCGCACAAAGGCTTTGCCACGTAATTACAGGCTCCTATTAAGTCGAGATCTGCCCTCCCAGGGGTCTCCAATTTTCTTGTATTCCCTACAAAGCCTCCTCTGCATGCCAGTTTGTGCCTTTTGAAGTGCCAGAGAGCTTCTTGATCCAACTGAGAAGGAAAAGGGAGCTCAGCGAGAAGAGGGGGAGAGAGCGAAGGGAACGGGGGAAACCCACCACCACCACCCTCCTTCGGACTCTTGGAGCCCCCCGCTTTTTTTTCCCCCTCCTTACGAAAAGTAAAGTGAGACTCCTGCTCTCCAATACATCTGCAAGACATCACCCTCTCCTCCTGAAACTTTAGTCACTCCTGAGAATCCACAGGAGTGCAGAGAGGGGGAACACGTTTTCTTGAAGATGTTTTAAAGCTGGAACAAGCCTTCTTCTGTTGGTGCTTGAACTCTTGCCTGGGAATAACTTTTTTAACCTAAAAAAAAAAAAAAAAAAAAAAACCCACTTTGATTCTTCTCTCCCACCCCTTCTTCTCTCTTCTTCTGTTTGCCTAACTCCCCCGCCCTGCTGGCCTCTCCTTTCCTCTCTCCCCCTTATTATTATTTTTAGTGCGTGCGTGTGGACGCTTTTGGAGAGCTGGAAGGGATTTTTTTTTTCTCTCTCTCTCCTGACTTGAACATAGGGTGACTTTTCATTTTATTTTTTTGGTGTGGATTATCTCTTTGGACCGCGCCGGACTTGGCCTCAGGAAAGCAACCGAGGCTTTCGAAAGCTGCTGGTGCAGAACTCTCTGCTCTTACAGAAGGGGGTCCCCCGCCCTCTTTCCACTTTTTTTTTGTTGTTGTTCTTCCCCTCCTTCTCTCTCTCTCTCTCTCCCTCTCTCTCTCCACTCCCCCCCTCTCTCTGCCCCACTCGGCTCCTCTCCCCCCTCGCGCCCACAGCGTTTGGTGTTGATTCGAGCGGGAAAGGGGGGTGGGTGGGATCGGAGGGGAAGACCATGACCTCCAGCTACGGGCACGTTCTGGAGCGGCAACCGGCGCTGGGCGGCCGCTTGGACAGCCCGGGCAACCTCGACACCCTGCAGGCGAAAAAGAACTTCTCCGTCAGTCACCTGCTAGACCTGGAGGAAGCGGGGGACATGGTGGCGGCGCAGGCGGACGAGAGCGTGGGCGAGGCGGGCCGGAGCCTACTGGAGTCGCCGGGACTCACCAGCGGCAGCGACACCCCGCAACAGGACAATGATCAACTGAACTCAGAGGAAAAGAAGAAAAGAAAGCAGCGGAGGAACAGGACAACCTTCAACAGCAGTCAGCTGCAGGCTTTGGAGCGTGTCTTTGAGCGGACACACTACCCAGATGCTTTTGTGCGAGAAGACCTTGCCCGCCGGGTGAACCTCACTGAGGCCAGAGTGCAGGTGTGGTTTCAGAACCGAAGAGCCAAATTCCGCAGGAATGAGAGAGCCATGCTGGCCAATAAAAATGCTTCCCTCCTCAAATCCTACTCAGGAGACGTGACTGCTGTGGAGCAGCCCATCGTACCTCGTCCTGCTCCAAGACCCACTGATTATCTCTCCTGGGGGACGGCCTCTCCGTACAGCGCCATGGCTACTTATTCTGCCACATGTGCCAACAATAACCCTGCACAGGGCATCAACATGGCCAACAGCATTGCCAACCTGAGACTGAAGGCCAAGGAATATAGTTTACAGAGGAACCAGGTGCCAACAGTCAACTGAGGAAAAAAAATAATTAAACAGGCCTAAGAAGAAATCAAAAACCATAAGACACCTATCCTGCTCTGTCATTTCTACATCTGCTGGGAAAAAAAAAATAAAAACAAACAAAAAAACCAGAACTAAACTATTGGGACCATGGCAGAGAAAAGCAGGAGAGGAGCAAAATGAAAATTAGTTAACAAATGTTCCTCCTCCCTCTGGGATACCACCACCACTTGTTTCTGTGTGTGTTAATTTTGTTTTTCCTTCTATTCATATGCTTTGCTTAATATACTCTGAGCTTCTTCAGTTAAGTTCAGCCCACCCACCCCCATGATTGTATGGGTTTTAAAAGAATCAACAGCAGCCAAAGAAACCATGTATGTATATATATATTCAGAATAATTGCCTGTAGTCTCCTCATTGACCTGTTTGAACCTCAGTGCCTTACCCCATCCTTCTTCCCAGTTCTCTGGACAGAAGCTCTAGGAACTTCTGAAAAGCCAAAGTCTTTCTGAAGAATTTGTGCCAGACATAATTCCCTTGCTCATTGTCTCCATTTCTGTTGGTTATGGTAAGGTTACTCCTTCAGCTACTATGGGGGAAAAAAAAAGCAATTGTATACAACATCTGGTCACTGGCCTATCTAATCCAATGTGATTGGCTATGTCTGGCTAATTCCAAGCTCTAAGCTCTAGATTTAAGCTCTAAGCAATAGAGGATAAAATCACCTCACTACTACCCCTCACCCCTATCCAAGCAGTCATCTTAAGTTAAAGATATTTGTTGTTTCTTGAGTAATTTGCTAACATAGGCTTATCTGATAGAAGACATATTTTTCACTTGAGAGAGGAAGAGACATTTCTCTAGGAAAACAAACACTAAGTTGTGAAAGGCATGGCCTTACATCTCTTTGGTGCCTTAAGGGTAGTCAGATGCACACTTATATATATACTGTATATATTTATATATTATATATATATATATATATATAAAATATTCTTGCAAGCTTGAGTTTGCAGTTTCTCAAACACTACCAAGAGCAAATTTCACACCATCACCAGTGTCCTTATTTCTACCGTGGTAAGACTTTTAATTAGGGATCTTGTTTCCTTTCTTTCTCTACACAAAATTCTCACTATGGCCACAGAGCAATTGATAAAGCAGCTGTCTGAGAACAGTTATAACTTTCACATTAGTGTTTTAAATAAATTAGAAGCAACTGAGGCTATGAAAATGTCCTTAAGTTTGGCACCTGAACTCTGGTGAAATGCTGATGCATACTAAACTATCATGAGAATTAGACTTAGCAGAGGAAAAGCCATTTAGTTATCTGTCCTTTTTCCACTCAACAGGAATACGAAAGATACATGATTCAGTGAAATACTTAAAGGTTTATCTAAATTCTAAGAATTTGAAAGGCAATTGTTGGCTATTGGTATCTTCTAATCTACTGAAATAAACTTTAGTATCTAGGGCTGGGAGCCAGGACTGACTGATCTTTTGTTTCTTCCTCTTTTGTTTCCACCCATGCTTTTGAAACTTGTCAGGTAGACATGACCAAACCACATTATCATGCTGTGCCTTGGTTTACTATTTTTTTTTTTTTTTTTTGTAAAATGGGTTTAACAATACTTACCTACCTCACAGGGGTGTTGTGAGGCTCTAATCATTTGCTCCTTTATCCTTTCCTGTATTCTCTGTATGTCCAGCACTTTGTAGCCATGGGAGGGAAGGGACTATGAAAGTATACTATGTTCATAGAAGGCTATGGTACCCAGAAGCCTTGTTTTCTAGCAAGGAGATGCTACCTTGCTGTAAATACTCATAACCGTGCATTTGGAAATGAGAAATAGGTTTATATTTGCAGACCTCTCAAAAATCACATCATTTGACCAAAAAATAACTTGAGACACACAGAACAGACATCTCTTTGACTTATATTTTCATCTTGACCAACTTGGATTTATATATAAATGGATTAAACTTGGATTTATAATAATTTTTTATATTAGTGAAAAGCTTTAGATTGATTTTGGCCAAATATGCCAACTGTGAGTAACAGGCAAGGATTTGGGACTTAAGCTCCACTTTCAGGACACATTTGCGATTTTCCCTGGCATATCAGGTTGAACTAATGGTGGTATTTTCAGTCCAACAGTCATCAATCTGAAAATGTATTTCCAAGGTTGATTCTAAACTTTTTTTTTAATAAGAAGGAAGCCCATCTTATATGCATTTTGCTTTCATCAACTGATTTCCTCTTCTTTTAACATACTATTAAATCATTTCTTACTGAATGGTTTATGTAGGCTGGCTGAACAACACACATTACTGCTTCCTATTCTTCAACTAATTCTCTTGTCCCACTAGCTGCTGAAGATTATCAAGTTTTTATCCTAACAGATTAAAACATTCTTTGTGAAAAGAGTGCAATCCAAGAAACTAGTGAACCTTTGGAGTTTGGGGAGAACAACTAAAAGAAGATTTGCGGTGTATAGTGCAGGGAATAGACATCATAGGTGAAAAGATTTTGCTAAAAATGGGGGGAATCTTAGAAGAGGGAGTAAAGAGATAGAGGATCATGTAAAACAAAGTCATTTGAAACTTTCGTCCTGACATTCATTTTGAGTGGTGAAGAGAAAAGCCTGAACCCCCATAACCCCAGTGCTGGATGAAATTAGGCAGTGCCTGGGCAAGTGCTTGCTGTCCAGGAATATCATAGAGTAATCAATACTATCAGTCATAGGAGCTTTTATTGCTAGTGAATCAATACCACAAACATCGACTATGTAAGGATCTGATGGAGAAATGTACACAGGGTACCAGCACTCAGTTTTGTTCATTCATTATTGATTTTTAAAAAATAGAAGTTTCATATGTGATTTGGAAGAACAGCATTCTGGCTCCTAAGCCAGGTGTGAGAGGGCCTCAGGGCCACAAAGTTCAAAGGCATATCCCTGTTCTTCATCATCTATACCCAGATAACAGATGAATACATGCCAACCGTCTGTGTTCCCAGTCCCTCCCCAAACAAGACACTGATTTGTAGAGTGCTTGGCAGGTTAAATCAAGCTGGAAGAGGTGACTGGATAAAAAAGGGAATGACATTTAGGGTATAAAGATCTCATAAGAAATGTAATATGTAAATTATATCTTGCTTTATGATGTAAAATATACATTGTTTGCGCTAGAATAGAAGTGATTCCTTTTCAATAAAAAGAAAGAAGGATACAACTA

**Putative binding sites for miR-140-3p were validated by RNAhybrid**


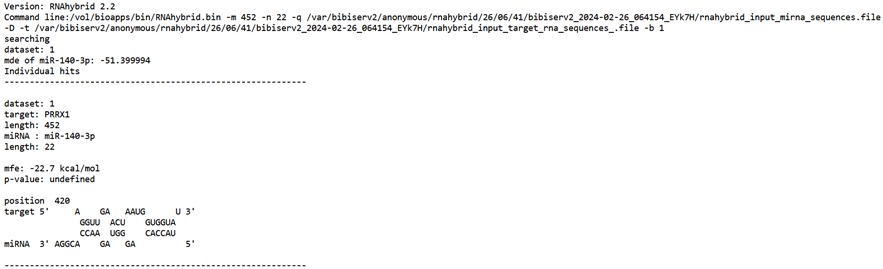


**WT 3'UTR of Prrx1 obtained by primer pmir-PRRX1-140-f and pmir-PRRX1-140-r**

gctagcTCCAGCACTTTGTAGCCATGGGAGGGAAGGGACTATGAAAGTATACTATGTTCATAGAAGGCTATGGTACCCAGAAGCCTTGTTTTCTAGCAAGGAGATGCTACCTTGCTGTAAATACTCATAACCGTGCATTTGGAAATGAGAAATAGGTTTATATTTGCAGACCTCTCAAAAATCACATCATTTGACCAAAAAATAACTTGAGACACACAGAACAGACATCTCTTTGACTTATATTTTCATCTTGACCAACTTGGATTTATATATAAATGGATTAAACTTGGATTTATAATAATTTTTTATATTAGTGAAAAGCTTTAGATTGATTTTGGCCAAATATGCCAACTGTGAGTAACAGGCAAGGATTTGGGACTTAAGCTCCACTTTCAGGACACATTTGCGATTTTCCCTGGCATATCAGGTTGAACTAATGGTG**GTA**TTTTCAGTCCAACtctaga

**Mutant 3'UTR of Prrx1 obtained by primer pmir-PRRX1-140-f and pmir-PRRX1-140m-r**

gctagcTCCAGCACTTTGTAGCCATGGGAGGGAAGGGACTATGAAAGTATACTATGTTCATAGAAGGCTATGGTACCCAGAAGCCTTGTTTTCTAGCAAGGAGATGCTACCTTGCTGTAAATACTCATAACCGTGCATTTGGAAATGAGAAATAGGTTTATATTTGCAGACCTCTCAAAAATCACATCATTTGACCAAAAAATAACTTGAGACACACAGAACAGACATCTCTTTGACTTATATTTTCATCTTGACCAACTTGGATTTATATATAAATGGATTAAACTTGGATTTATAATAATTTTTTATATTAGTGAAAAGCTTTAGATTGATTTTGGCCAAATATGCCAACTGTGAGTAACAGGCAAGGATTTGGGACTTAAGCTCCACTTTCAGGACACATTTGCGATTTTCCCTGGCATATCAGGTTGAACTAATGGTG**CAT**AAAAGTCAGGTACtctaga
