# Supplementary figures and images for: Reciprocal negative feedback between Prrx1 and miR-140-3p regulates rapid chondrogenesis in the regenerating antler
Source: Cell Mol Biol Lett. 2024 Apr 20;29:56. doi: 10.1186/s11658-024-00573-x (PMC11031908; doi:10.1186/s11658-024-00573-x)

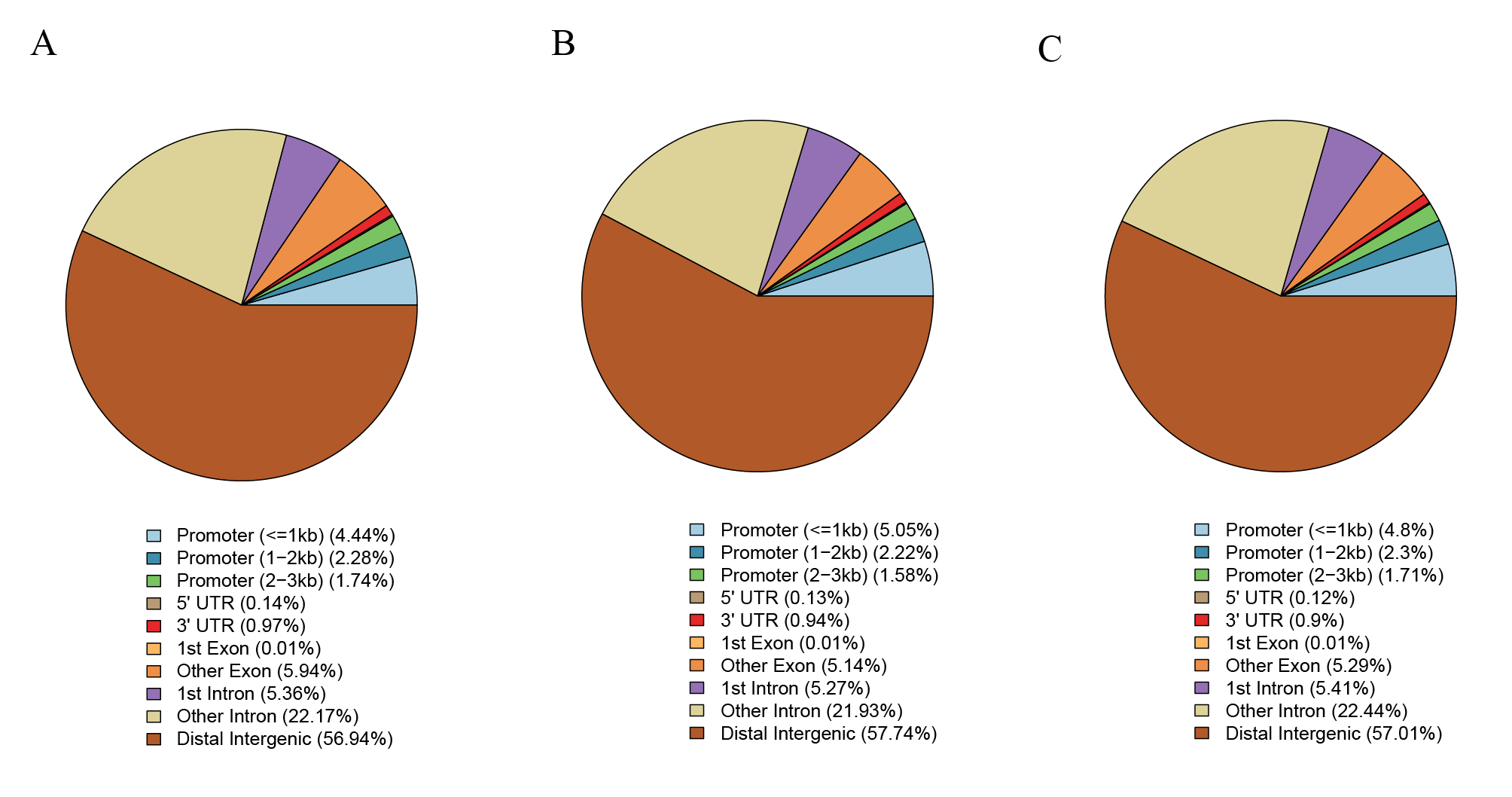

Supplement: Supplementary file 8 — Additional file 8: Supplementary Fig. 1. Genomic annotation of the ATAC peaks. A RM ATAC peaks. B PC ATAC peaks. C CA ATAC peaks. [file 11658_2024_573_MOESM8_ESM.tif]

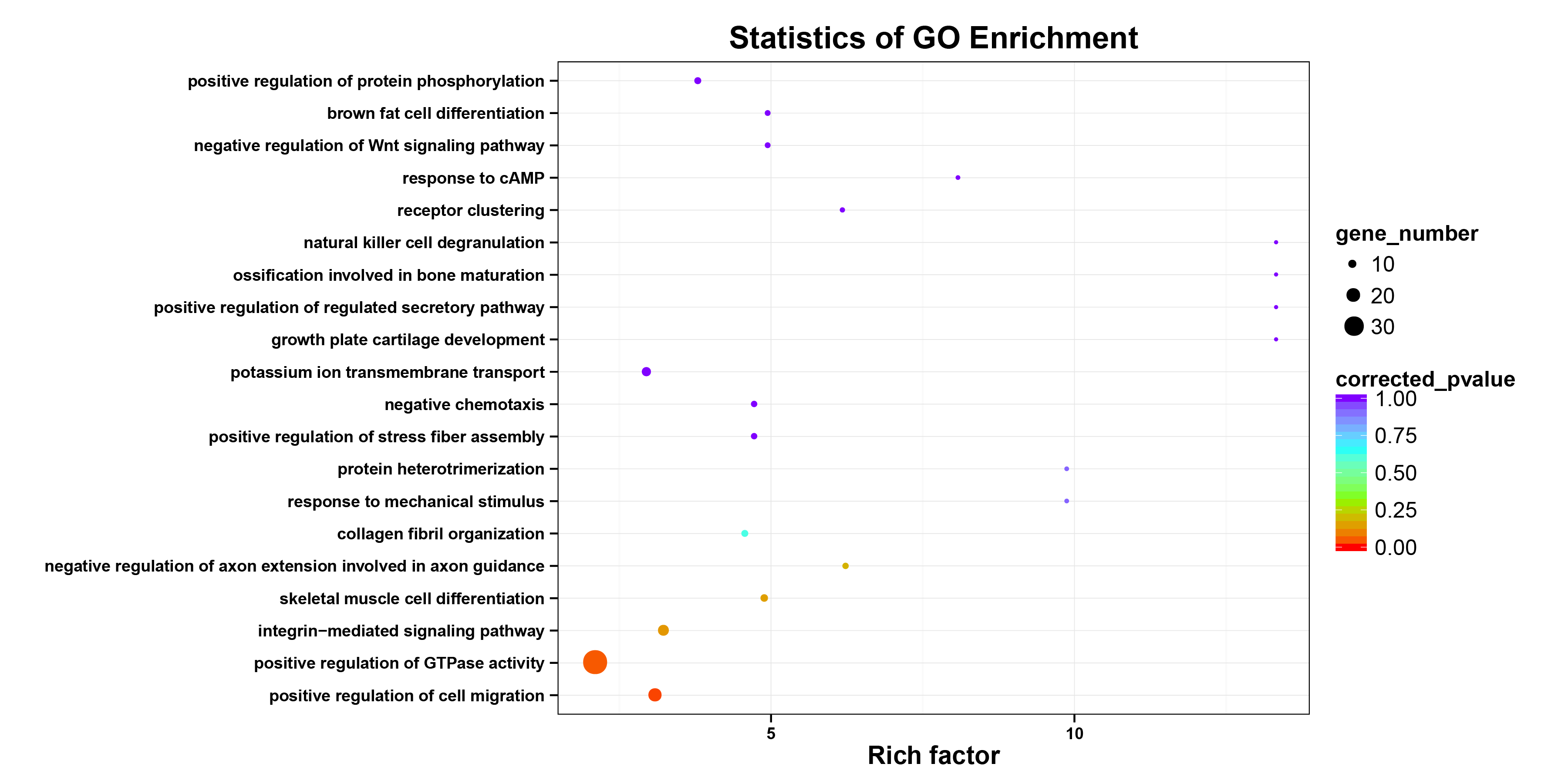

Supplement: Supplementary file 9 — Additional file 9: Supplementary Fig. 2. GO enrichment of the corresponding genes in cluster 3 of ATAC peaks. [file 11658_2024_573_MOESM9_ESM.tif]

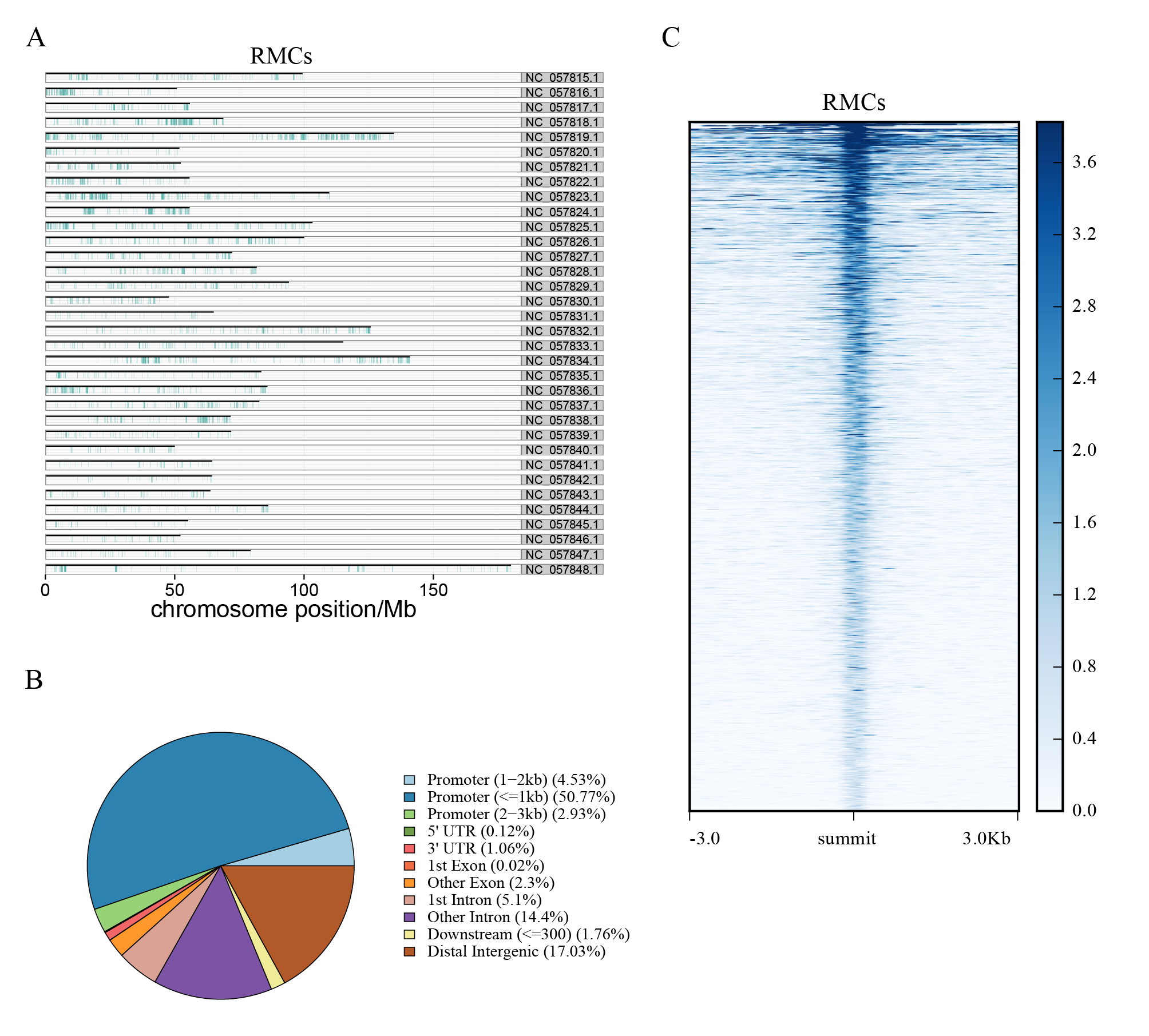

Supplement: Supplementary file 10 — Additional file 10: Supplementary Fig. 3. Prrx1c CUT&Tag-seq. A Prrx1 CUT&Tag peak on chromosome distribution. B Prrx1 CUT&Tag narrow peaks plot. C Prrx1 CUT&Tag summits heatmap. [file 11658_2024_573_MOESM10_ESM.tif]

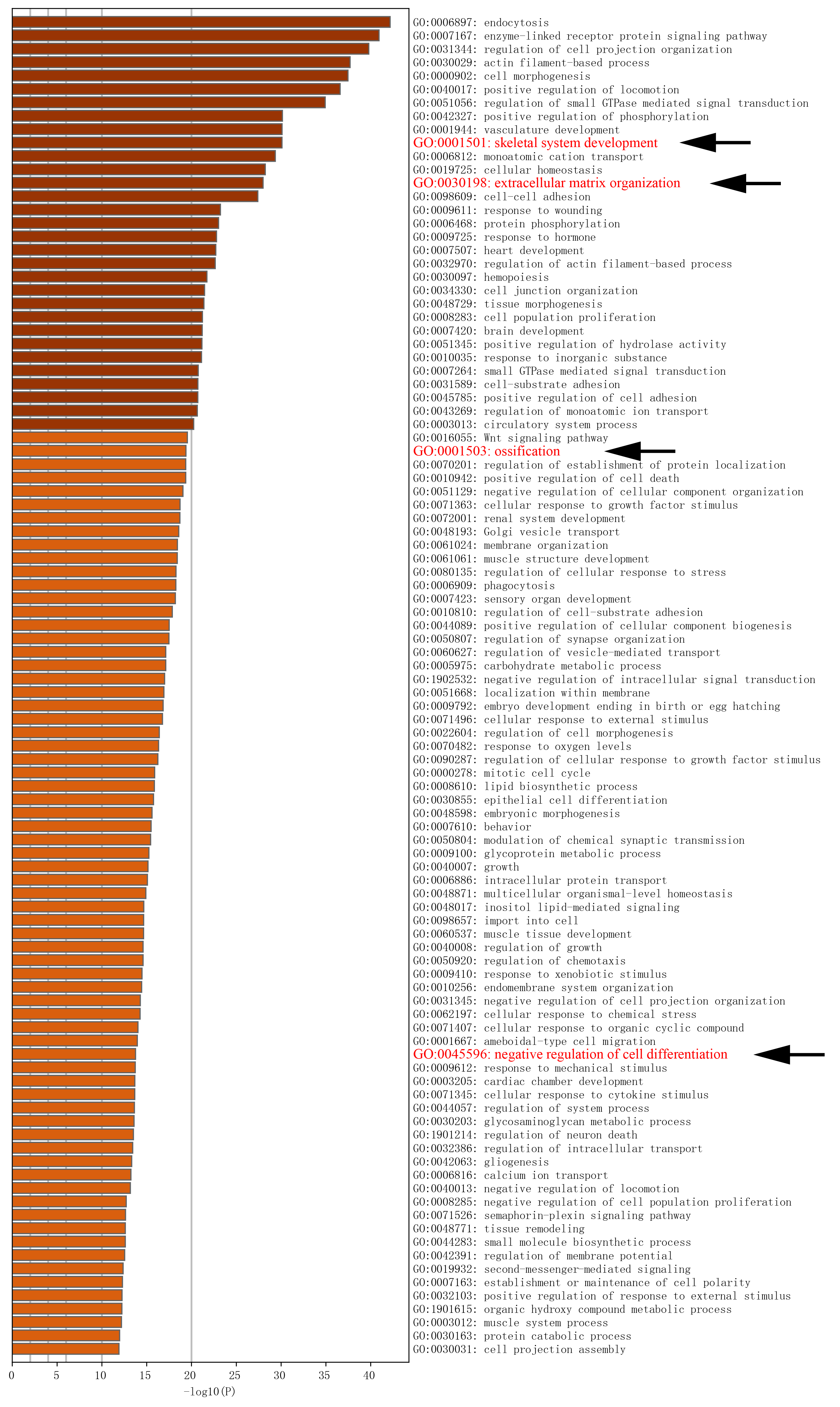

Supplement: Supplementary file 11 — Additional file 11: Supplementary Fig.4. GO enrichment of target genes of miR-140-3p. [file 11658_2024_573_MOESM11_ESM.tif]
